# Transcriptomics reveal useful resources for examining fruit development and variation in fruit size in *Coccinia grandis*

**DOI:** 10.3389/fpls.2024.1386041

**Published:** 2024-05-28

**Authors:** Mitrabinda Panda, Seema Pradhan, Pulok K. Mukherjee

**Affiliations:** ^1^ Biotechnology Research Innovation Council-Institute of Life Sciences (BRIC-ILS), Bhubaneswar, India; ^2^ Regional Centre for Biotechnology, Faridabad, India; ^3^ Biotechnology Research Innovation Council-Institute of Bioresources and Sustainable Development (BRIC-IBSD), Imphal, India

**Keywords:** *Coccinia grandis*, fruit development, differential gene expression, simple sequence repeats, Ivy gourd

## Abstract

**Introduction:**

The Cucurbitaceae family comprises many agronomically important members, that bear nutritious fruits and vegetables of great economic importance. *Coccinia grandis*, commonly known as Ivy gourd, belongs to this family and is widely consumed as a vegetable. Members of this family are known to display an impressive range of variation in fruit morphology. Although there have been studies on flower development in Ivy gourd, fruit development remains unexplored in this crop.

**Methods:**

In this study, comparative transcriptomics of two Ivy gourd cultivars namely “Arka Neelachal Kunkhi” (larger fruit size) and “Arka Neelachal Sabuja” (smaller fruit size) differing in their average fruit size was performed. A *de novo* transcriptome assembly for Ivy gourd was developed by collecting fruits at different stages of development (5, 10, 15, and 20 days after anthesis i.e. DAA) from these two varieties. The transcriptome was analyzed to identify differentially expressed genes, transcription factors, and molecular markers.

**Results:**

The transcriptome of Ivy gourd consisted of 155205 unigenes having an average contig size of 1472bp. Unigenes were annotated on publicly available databases to categorize them into different biological functions. Out of these, 7635 unigenes were classified into 38 transcription factor (TF) families, of which Trihelix TFs were most abundant. A total of 11,165 unigenes were found to be differentially expressed in both the varieties and the *in silico* expression results were validated through real-time PCR. Also, 98768 simple sequence repeats (SSRs) were identified in the transcriptome of Ivy gourd.

**Discussion:**

This study has identified a number of genes, including transcription factors, that could play a crucial role in the determination of fruit shape and size in Ivy gourd. The presence of polymorphic SSRs indicated a possibility for marker-assisted selection for crop breeding in Ivy gourd. The information obtained can help select candidate genes that may be implicated in regulating fruit development and size in other fruit crops.

## 1 Introduction

Fruit is a characteristic feature of angiosperms and can be a chief source of vitamins, fibres, carbohydrates and other nutrients necessary for human diet (Zhao et al., 2021). Fruits in flowering plants usually develop from matured ovary or in some cases, accessory floral tissues contribute in fruit formation (Seymour et al., 2011; Zhao et al., 2021). In addition to being the edible part of most fruit-bearing plants, they also help in seed dispersal after maturation (Azzi et al., 2015), thereby allowing the successful adaptation of angiosperms on Earth. Fruit has diverse morphology in order to cope with changing environments (Wang et al., 2022) and usually undergoes three distinct stages of development; fruit set, fruit growth, and fruit maturation (Azzi et al., 2015). In the first stage of development, the ovary’s growth is followed by successful fertilization, which leads to the decision for fruit setting. In the second stage of fruit development, fruit grows due to cell division. The formation of seed and embryo development takes place at this stage. Hereafter, the fruit cells don’t divide, rather the fruit increases its volume by cell expansion. This stage is the longest and decides the size of a fruit. The third stage is known as fruit maturation where deposition of different types of storage products in fruits occurs and at this stage, fruit size increases very slowly (Gillaspy et al., 1993; Mu et al., 2009; Zhao et al., 2021).

Fruit size and shape constitute important agronomic traits and also contribute to plant fitness in the course of evolution (Hussain et al., 2020). Fruits that are larger in size and weight are usually preferred by consumers, lending them greater economic value (Huang et al., 2023). Many external and internal factors influence fruit size including genetics, environment, cultivation practices etc. and have been studied in various reports (Huang et al., 2023; Jahed and Hirst, 2023). Apart from these, molecular mechanisms such as cell division, cell expansion, and endoreduplication, play a vital role in determining fruit size and shape (Chevalier et al., 2014; Okello et al., 2015; Mauxion et al., 2021). More detailed analyses have revealed the role of phytohormones, transcription factors, quantitative trail loci (QTLs), miRNAs, and signalling pathways in fruit development (Guo et al., 2010; Libault et al., 2010; Xu et al., 2013; Hussain et al., 2020; Huang et al., 2023). Nowadays, developing plants with gene knock-out and knock-in is feasible due to advanced technologies for genome editing and plant transformation protocols. Hence the only challenge is to identify genes and regulatory elements controlling fruit sizes that can be later implemented for crop improvement through genome editing and through marker-assisted selection (Hussain et al., 2020).

Ivy gourd (*Coccinia grandis)* belongs to the Cucurbitaceae family and is a nutrient-rich vegetable found in sub-Saharan Africa, tropical Asia, neo-tropics, and India (Mohanty et al., 2017). The cucurbitaceae family consists of many members that produce a variety of fruits and vegetables for everyday consumption. Great variations have been observed for fruit sizes in this family which comprises of many important vegetables such as pumpkin and bottle gourd (Xanthopoulou et al., 2017; Zhang et al., 2020). Such morphological differences in fruits of the same lineages can be used to understand the genetic entity that controls the fruit size. We have selected two varieties of Ivy gourd namely “Arka Neelachal Kunkhi” and “Arka Neelachal Sabuja”, which have considerable differences in fruit size and shape, with “Arka Neelachal Kunkhi” being elongated in shape and “Arka Neelachal Sabuja” being ovoid shaped. “Arka Neelachal Kunkhi” is a high yielding variety that produces around 800 fruits per season with yield potential of 15–20 tonnes/ha. It is a striped Ivy gourd consumed both in raw (in salads) as well as in cooked form. On the other hand, “Arka Neelachal Sabuja” is a vigorously growing plant that yields 70–80 harvests per season with a yield potential of 20–25t/ha (https://www.iihr.res.in/central-horticultural-experiment-stationches-bhubaneswar). The fruits are consumed widely in India and are a good source of several vital minerals and vitamins. Therefore, analyzing the factors associated with fruit morphology at a molecular level will be helpful in future gene-editing efforts.

Various genes regulating phytohormones such as auxin, gibberellins, cytokines, abscisic acid and ethylene are known to regulate fruit development in plants. For example, over expression of *GmYUCCA5* gene resulted in short siliques in *Arabidopsis* (Wang et al., 2017). Various transcription factor families such as B3 family, *YABBY* family, Zinc finger family are known to regulate fruit sizes. In addition to that, microtubule play an active role in cell division and hence play a role in fruit size determination (Hussain et al., 2020). With the progress in high-throughput sequencing techniques with computational, statistical methods and development of multi-omics, comparative transcriptomics has become a norm for identification of key genes underlying important biological phenomena such as control of fruit growth and size (Peng et al., 2022; Jahed and Hirst, 2023). Transcriptomic analysis can reveal genes regulating important agronomic traits in plants and can also reveal differences in gene expression in response to environmental factors (Zhang et al., 2020). The method has been used in numerous studies where the researchers have identified genes based on their differential expression in various tissues and developmental stages of fruits in the cucurbits (Xanthopoulou et al., 2017; Abbas et al., 2020; Zhang et al., 2020; Luo et al., 2021; Xanthopoulou et al., 2021). The present study aims to add to the limited collection of genes and regulatory elements that govern fruit development and morphology in cucurbits. Here we have generated the transcriptome of two cultivars of Ivy gourd (“Arka Neelachal Kunkhi” and “Arka Neelachal Sabuja”) with different fruit sizes and to identify genes, regulators, and key pathways involved in fruit development and size regulation. Information obtained will be very useful for re-architecting the shape and size of fruit using gene-based technology.

## 2 Materials and methods

### 2.1 Plant material

Two varieties of *C. grandis* with contrasting fruit shape and size were collected from Central Horticultural Experiment Station (CHES), Bhubaneswar (20.2446°N, 85.7812°E). Seedlings of *C. grandis* var. “Arka Neelachal Kunkhi” (Long, slender fruit) and *C. grandis* var. “Arka Neelachal Sabuja” (Short, oval fruit) were grown in the fields at ILS, Bhubaneswar and flowers were tagged on the day they opened completely. Fruits were collected at four different stages of development: 5 Days after anthesis (DAA), 10 DAA, 15 DAA and 20 DAA, for both the varieties of *C. grandis*. Leaf tissue was also collected from both plants to use for the generation of transcriptome to generate better coverage for the transcriptome which will allow better assembly. All samples were collected in triplicates (biological), snap-frozen in liquid N2, and stored at -80°C till further processing.

Fresh fruits were used for morphological studies including measurements of fruit length and width. Anatomical studies were also performed through the observations of thin cross sections from different stages of fruit development in both the varieties under light microscope (Model Stemi508, Make Zeiss) and Scanning electron microscope (SEM - FESEM-EDX, Model JSM-IT800, Make JEOL). The SEM images (Scale 10 µm) were exported to the ImageJ software for cell number count and area measurement in µm^2^. These steps were performed with three biological replicates.

### 2.2 RNA isolation and RNA-Seq

Total RNA was extracted from 100mg of frozen plant tissues using a Nucleospin Plant and Fungi RNA extraction kit (REF 740120.50, Macherey-Nagel, Düren, Germany) following the manufacturer’s protocol. The quantity and quality of the RNA were analyzed on 1% agarose gel, and Qubit 4.0 fluorometer (Thermo Fisher Scientific, USA) and an Agilent TapeStation system (Agilent, USA). A total of 1μg of total RNA with RNA integrity number (RIN) > 7 was used for library preparation. The libraries were prepared taking three biological replicates of each sample (fruits of 5 DAA, 10 DAA, 15 DAA, 20 DAA, young leaves) and using the TruSeq Stranded mRNA Library Prep kit (Illumina, San Diego, USA) following the manufacturer’s instructions. Library quality was evaluated using (Thermo Fisher Scientific, USA) and Agilent TapeStation system (Agilent, USA). After quality assessment, a total of thirty libraries representing three biological replicates each of five samples of “Arka Neelachal Kunkhi” (hereafter depicted as “ANKu”) and “Arka Neelachal Sabuja” (hereafter depicted as “ANSa”) were pooled and paired-end sequenced on the NextSeq550 (2x150) platform (Illumina, USA) at Institute of Life Sciences, Bhubaneswar, Odisha.

### 2.3 Processing of reads and transcriptome assembly

A total of 405 million reads in “ANKu” and 566 million reads in “ANSa” were obtained from all the libraries after demultiplexing with bcl2fastq software (Illumina, USA). The raw reads have been submitted to NCBI SRA database with the BioProject ID PRJNA851184. The reads were screened for quality and trimmed with Trimmomatic v. 0.39 with the following parameters: (ILLUMINACLIP: TruSeq3- PE2.fa:2:30:10 LEADING:5 TRAILING:5 SLIDINGWINDOW:5:10 MINLEN:50). The high-quality reads after removal of adapter sequences were used for *de novo* assembly using the Trinity pipeline (version 2.11) with the default parameters (Haas et al., 2013). CD-HIT-EST (Li and Godzik, 2006) was performed at 98% similarity to remove redundant contigs.

### 2.4 Assembly quality assessment and annotation

The quality of the *de novo* transcriptome assembly was assessed in two ways (i) by calculating the percentage of reads mapped onto the assembled unigenes and (ii) by using the curated datasets at BUSCO (Simão et al., 2015). For the first method, the filtered reads from each sample were mapped onto the assembled unigenes using Bowtie2 (Langmead and Salzberg, 2012) and percentage of mapped reads were plotted in a graph on MS Excel. The BUSCO analysis was performed using the eudicot_odb10 data set (Simão et al., 2015).

Functional annotation was carried out using Blastx against various databases including UniRef90 database (https://www.uniprot.org/help/downloads) Uniprot-SwissProt database (https://www.uniprot.org/), COG (https://ftp.ncbi.nlm.nih.gov/pub/COG/COG2020/data/), KEGG (https://www.genome.jp/kegg/kaas/), Pfam (http://pfam.xfam.org/). Venn diagrams were generated using the tools provided at https://bioinformatics.psb.ugent.be/webtools/Venn/. GO terms were assigned to the unigenes after blastx with Uniprot Swissprot database and all relevant datasets for the annotation were downloaded from http://geneontology.org/, as described in an earlier study (Shyamli et al., 2021).

### 2.5 Transcription factor family identification and SSR mining

Transcription factors were identified in the Ivy gourd transcriptome in two stages. In the first pass, the HMM profiles of all available gene families were downloaded from Pfam (http://ftp.ebi.ac.uk/pub/databases/Pfam/current_release/Pfam-A.hmm.gz) and used to search for the conserved Hidden Markov Model (HMM) profiles in the peptide sequences of Ivy gourd. However, the HMM profiles for all the transcription factor families were not present in the Pfam database. So, in a second pass of TF prediction, peptide sequences for individual TF families of Arabidopsis were downloaded from TAIR and aligned to generate HMM profiles using HMMER v. 3.3 (http://hmmer.org/). These profiles were then used to search for TF families in the peptide sequences of Ivy gourd.

Simple sequence repeats (SSRs) were identified in the unigenes using MISA software (Beier et al., 2017) with the following parameters: Mono-nucleotides 10 repeats, di-nucleotide 8, tri- and tetra-nucleotide 4 repeats, penta-nucleotide and hexa-nucleotide 3 repeats. A total of 20 SSR markers associated with differentially expressed genes were randomly selected having a length of 15bp to 24bp. Primers for genic SSRs sequences were designed by using MISA web tool (https://webblast.ipk-gatersleben.de/misa/index.php?action=1) based on the following criteria; a G/C content between 40% and 70%, an annealing temperature between 54°C to 63°C, minimum product length of 100bp and primer length 10–24 nucleotides. All the candidate SSR primer pairs were synthesized by Eurofins Scientific India Pvt Ltd (Bengaluru, India). The PCR reactions were conducted with 25ul reaction volume, and following conditions: initial denaturation at 95°C for 3min followed by 30 cycles of denaturation at 95°C for 30sec, annealing at 54°C for 45sec and extension at 72°C for 45 sec. The final extension was done at 72°C for 5min and hold at 4°C. PCR amplifications were performed using genomic DNA isolated from 15 DAA stage of fruit (using NucleoSpin™ Plant II DNA extraction kit, Macherey-Nagel) as template and the PCR products were verified with 3% metaphor agarose (Lonza, Basel, Switzerland) and 100bp DNA ladder (GeNei™, Bangalore, India).

### 2.6 Differential gene expression analysis, GO term enrichment and biological pathway analysis

*In silico* gene expression analysis was carried out as described by (Suranjika et al., 2022). Briefly, we used Bowtie2 (Langmead and Salzberg, 2012) to map the short reads from all sample libraries (including replicates), onto the assembled transcriptome and abundance was estimated by RSEM i.e RNA-Seq by Expectation-Maximization (Parrish et al., 2011). Empirical Analysis of Digital Gene Expression (EdgeR) was used to obtain differentially expressed unigenes in Ivy gourd as well as to normalize the expected counts for relative expression (Robinson et al., 2010) with FDR correction ≤ 0.05, *P* value ≤ 0.001, fold change ≥ 2. The normalization factor of effective library size was performed by Trimmed Mean of M- values (TMM). Finally, the unigenes with threshold FDR ≤ 0.05 and log fold change |log_2_ FC| (≥ 1) were considered to be differentially expressed and selected for further analysis.

### 2.7 Quantitative real time PCR analysis

In order to check the expression of few genes to relate their roles in fruit size regulation, qRT- PCR analysis was performed by using *C. grandis* 18S rRNA as endogenous control to normalize the gene expression. Primers were designed by using the Primer ™Quest tool of Integrated DNA technology (IDT) software (**Supplementary Table S1**). Total RNA of all the stages of developing fruits (5 DAA, 10 DAA, 15 DAA, and 20 DAA) and with three biological replicates was isolated. First strand cDNA synthesis was accomplished by using 1μg of freshly isolated RNA using First strand cDNA synthesis kit (ThermoFisher Scientific, Waltham, MA, USA) following the specifications of the manufacturer. The cDNA products were diluted five times (1:5) and stored in -20°C. For Real Time PCR, each sample reaction was set up in a reaction mixture of 10μl containing 5μl of 2X GoTaq qPCR Master mix (Promega Corporation, Madison, WI, USA), 1 μl of gene-specific primer mix, 0.5 μl of 1:5 diluted cDNA and nuclease-free water for volume adjustments (Promega Corporation, Madison, WI, USA). The qRT-PCR assay was performed using the QuantStudio5 system (Applied Biosystems, ThermoFisher Scientific, Waltham, MA, USA), with the following reaction conditions; 95°C for 10min (initial denaturation) followed by 35 cycles of 95°C for 15s and 55°C for 1min. A melt curve program was performed by initiating at 95°C for 15s and reducing the temperature by 1.6°C/s until it reaches 60°C for 1 min followed by an increase in 0.15°C/s until it reaches 95°C. The relative fold changes were calculated by using the comparative 2^-ΔΔCt^ method (Livak and Schmittgen, 2001). The statistical analysis was done by calculating the standard deviation and the level of significance was determined by the Student’s t-test (*P ≤* 0.05).

## 3 Results

### 3.1 Morphological and anatomical characterization of Ivy gourd var “Arka Neelachal Kunkhi” and “Arka Neelachal Sabuja”

**Figure 1:**
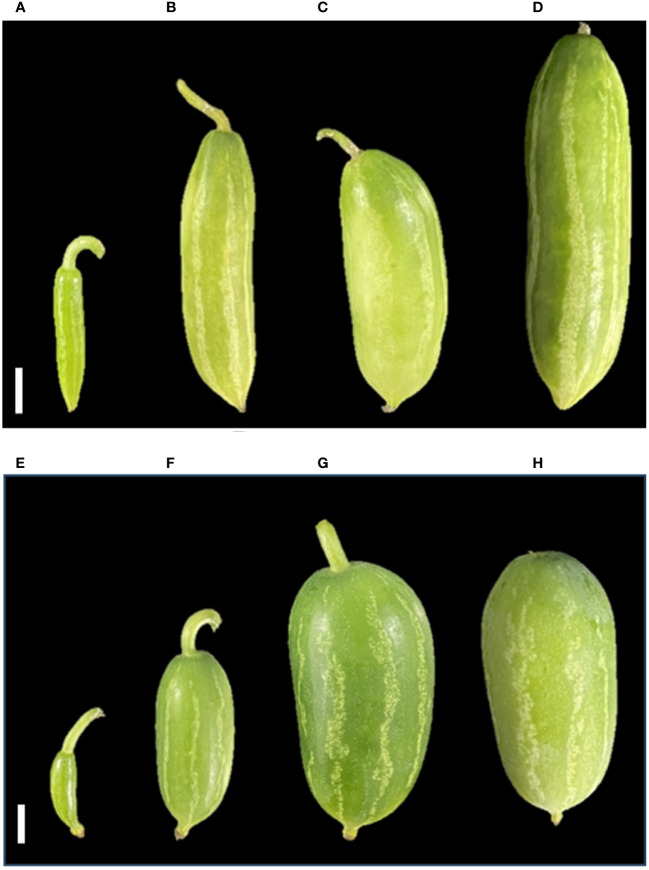
Morphological characterization of Ivy gourd var ANKu and ANSa at different stages of development; **(A–D)** ANKu fruit samples 5 DAA, 10 DAA, 15 DAA, 20 DAA. **(E–H)** ANSa fruit samples 5 DAA, 10 DAA, 15 DAA, 20 DAA; Scale bar 1 cm.

**Figure 2:**
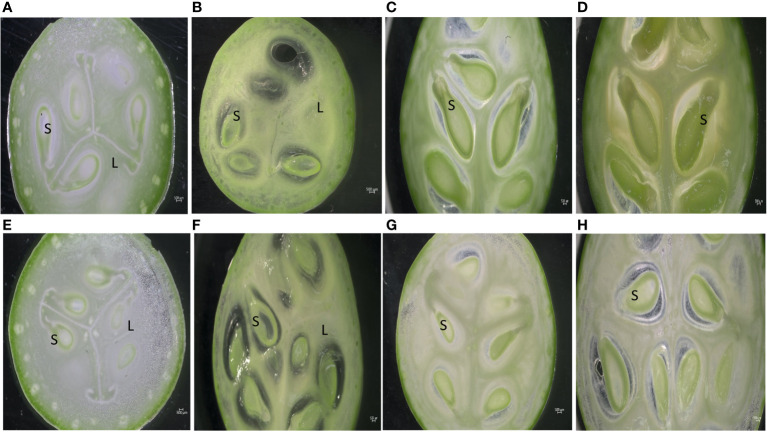
Anatomical cross-section of Ivy gourd var ANKu and ANSa at different stages of fruit development; **(A–D)** ANKu fruits at 5 DAA, 10 DAA, 15 DAA, 20 DAA. **(E–H)** ANSa fruits at 5 DAA, 10 DAA, 15 DAA, 20 DAA; (S – Seed, L – Locule); Scale Bar 500µm.

**Figure 3:**
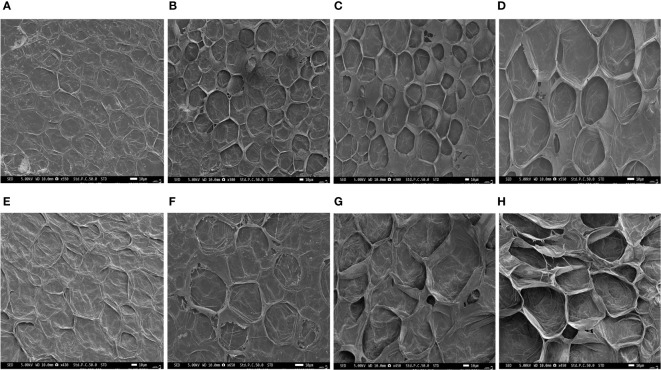
SEM micrographs of the anatomical cross-section of Ivy gourd var ANKu and ANSa at different stages of fruit development; **(A–D)** ANKu fruits at 5 DAA, 10 DAA, 15 DAA, 20 DAA. **(E–H)** ANSa fruits 5 DAA, 10 DAA, 15 DAA, 20 DAA; Scale bars 10 µm.

Phenotypic observations of two varieties “Arka Neelachal Kunkhi” (ANKu) and “Arka Neelachal Sabuja” (ANSa) showed remarkable size and shape differences in all the developmental stages taken in the present study (**Figure 1**). Two fruit variables such as length and width from all the collected samples (5 DAA, 10 DAA, 15 DAA, 20 DAA) were measured. At maturity, the mean length of ANKu (20 DAA) was found to be 9.02 cm and that of ANSa was 6.7 cm. Likewise, the diameter or mean width of ANKu and ANSa were measured as 7 cm and 8.92 cm respectively (**Supplementary Table S2**). Anatomical studies of fruits at each stage showed that the locule of ANSa expands gradually in every growing stage and the number of seeds in mature ANSa fruits is higher in comparison with ANKu (**Figure 2**). More detailed studies using SEM also showed differences in cell number in different stages of fruit development in both varieties (**Figure 3**). More number of cells were observed in ANKu when compared to ANsa. The cell numbers were observed to increase rapidly during the early stages of fruit development (5 DAA, 10 DAA) in both the varieties of *C. grandis.* However, this rapid increase stagnated as the fruits reached maturity and no rapid increase in number was observed (**Supplementary Figure S1A**). The number of cells were found to be higher in ANKu than in ANSa. To examine the cell expansion in both varieties, the cell area of different developmental stages was measured. Interestingly, we observed, a larger cell area in ANSa than in ANKu (**Supplementary Figure S1B**). Moreover, maximum expansion of cells was observed in 10 DAA to 20 DAA fruits of ANSa.

### 3.2 Assembly and annotation of the Ivy gourd transcriptome

A total of 971 million reads were obtained after short read sequencing of the two varieties of Ivy gourd. These reads were filtered to remove low quality reads and adapter contamination to obtain 948 million reads, giving us 96% retention of high-quality reads on average (**Supplementary Table S3**). A total of 155205 unigenes remained after processing, with an N50 value of 1472 bp (**Table 1**). During quality assessment, we observed that on average, 97.6% of the RNA-seq reads could be mapped onto the assembly (**Supplementary Figure S2A**). Analysis using the conserved orthologs in BUSCO database showed that 95.4% of complete BUSCOs from eudicots_odb10 database were represented in the Ivy gourd transcriptome (**Supplementary Figure S2B**).

**Table 1:** Assembly statistics for the transcriptome of Ivy gourd.

Attribute	Value
Total number of unigenes	155205
N50	1472 bp
Maximum length of unigene	17191 bp
Average length of unigene	818.58 bp

**Figure 4:**
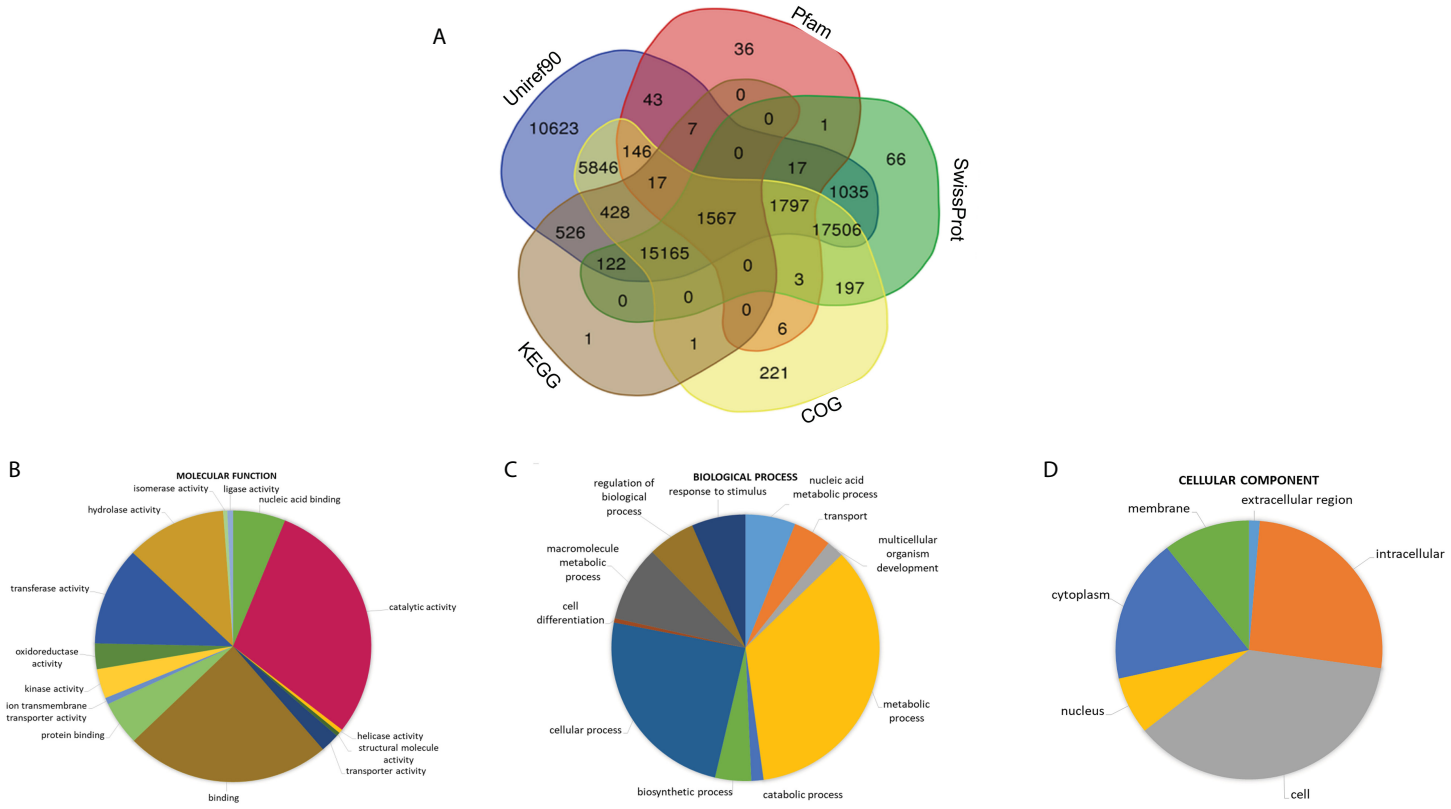
Functional annotation; **(A)** Venn diagram representing the annotation of protein coding sequences of *C. grandis* with various publicly available databases (Uniref, Pfam, COG, KEGG, Swissprot), GO terms classified into three major classes namely Biological Process, Molecular function and Cellular Components **(B–D)**.

We annotated the transcriptome of Ivy gourd against various databases and found that only 55,377 unigenes could be annotated, which means that only ~36% of the unigenes could find a match in any of the databases (**Figure 4A**). Using the results of Blastx against the Uniprot-Swissprot database, we assigned GO terms to the unigenes and found categories like “metabolic process” and “cellular process” were over-represented under the term “Biological Process” while categories like “catalytic activity” and “binding” accounted for the majority of unigenes under the “Molecular Function term” (**Figures 4B–D**). Transdecoder tool (https://github.com/TransDecoder/TransDecoder) predicted 47,296 complete CDS in the transcriptome of Ivy gourd which accounts for 30% of the total unigenes.

### 3.3 Differential gene expression analysis

**Figure 5:**
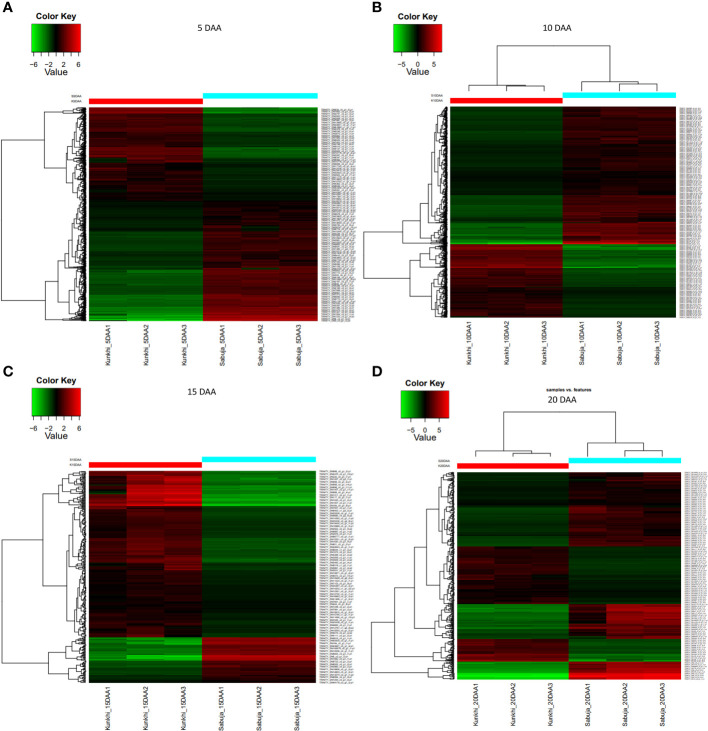
**(A–D)** Differential expression of Unigenes in ANKu and ANSa varieties of Ivy gourd: The figure depicts DEUs in the two varieties of Ivy gourd at **(A)** 5, **(B)** 10, **(C)** 15 and **(D)** 20 DAA of fruit development respectively. The analysis was performed taking ANKu (Kunkhi) as the reference and all expression analyses were performed using the pipeline provided in Trinity software package. The scale represents log2 –transformed mean-centered FPKM values generated with edgeR.

**Figure 6:**
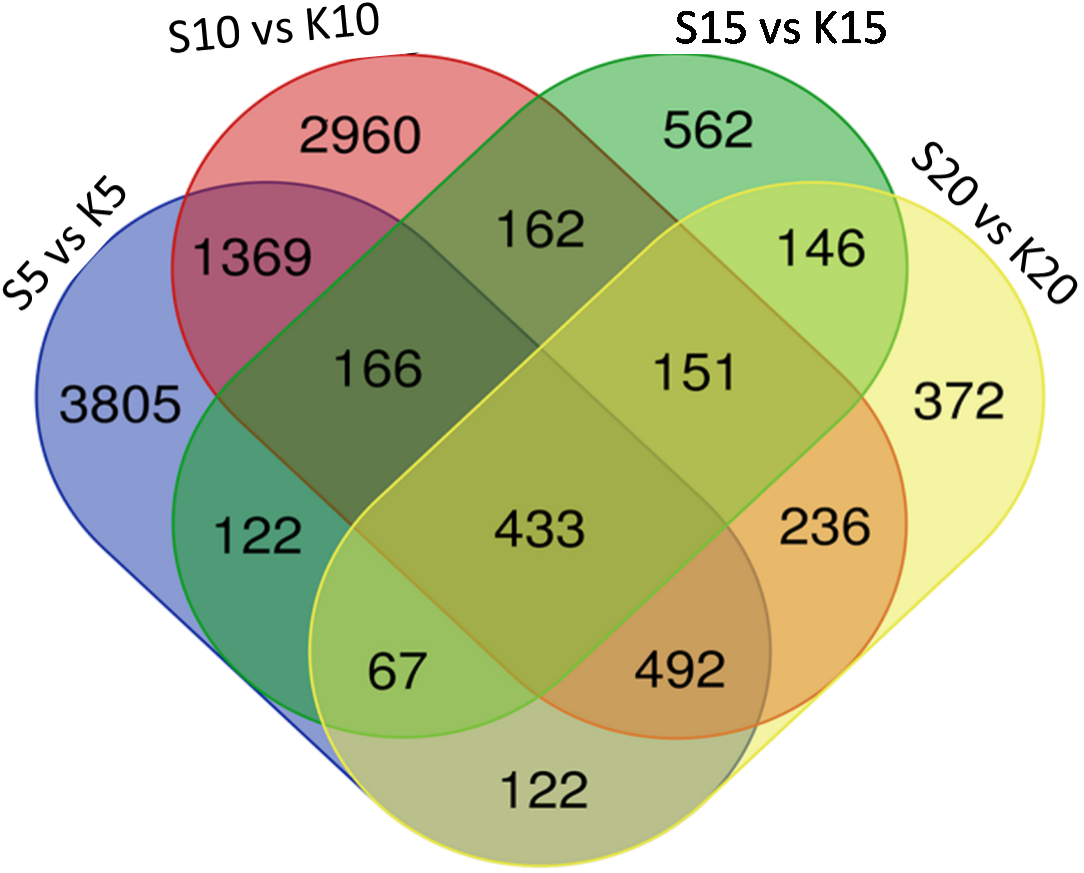
A comparative analysis of differentially expressed unigenes in both the varieties at each stage of fruit development. S refers to ANSa and K refers to ANKu in the Venn diagram which was created using the webtool at https://bioinformatics.psb.ugent.be/webtools/Venn/.

We generated expression profiles for the entire transcriptome after comparing the fruits from the two varieties at individual developmental stages i.e at 5, 10, 15 and 20 DAA (**Figures 5A–D**; **Supplementary Figure S3**). We found that at 5 DAA, compared with ANKu, fruits of ANSa showed up-regulation of genes encoding U-box domain containing proteins, F-box proteins, ABC transporter, MYB and NAC TFs among others and downregulation of calmodulin binding proteins, cyclin, serine/threonine protein kinase coding genes. At 10 DAA, genes coding for ROS scavenging, autophagy related proteins, certain transposons and TFs like WRKY and B3 were upregulated in ANSa while those for TFs like ERF, Trihelix and MYB were downregulated. There was a drastic shift in gene expression patterns during 15 DAA where majority of the unigenes were downregulated in ANSa as compared with ANKu. These included genes like DExH box, UDP glycosyltransferase, MADS box, Pentatricopeptide containing proteins etc. On the other hand, endochitinase encoding genes, ERF TFs and flowering time control proteins were upregulated in ANSa as compared to ANKu at 15 DAA. It was also interesting to observe that there were a number of unigenes coding for genome polyprotein in Papaya ring spot virus (POLG), that were downregulated in ANSa variety. The expression profile at 20 DAA showed that POLG coding unigenes were differentially regulated in ANSa with most of them being upregulated while genes like Cytochrome P450, MYB TFs were downregulated (**Supplementary Table S5**). A comparison of the DEUs at each stage of fruit development revealed 433 unigenes that were common to all stages (**Figure 6**). These comprised of genes coding for MYB TF, heat stress transcription factor, trihelix TF, UDP-glycosyltransferase etc.

**Figure 7:**
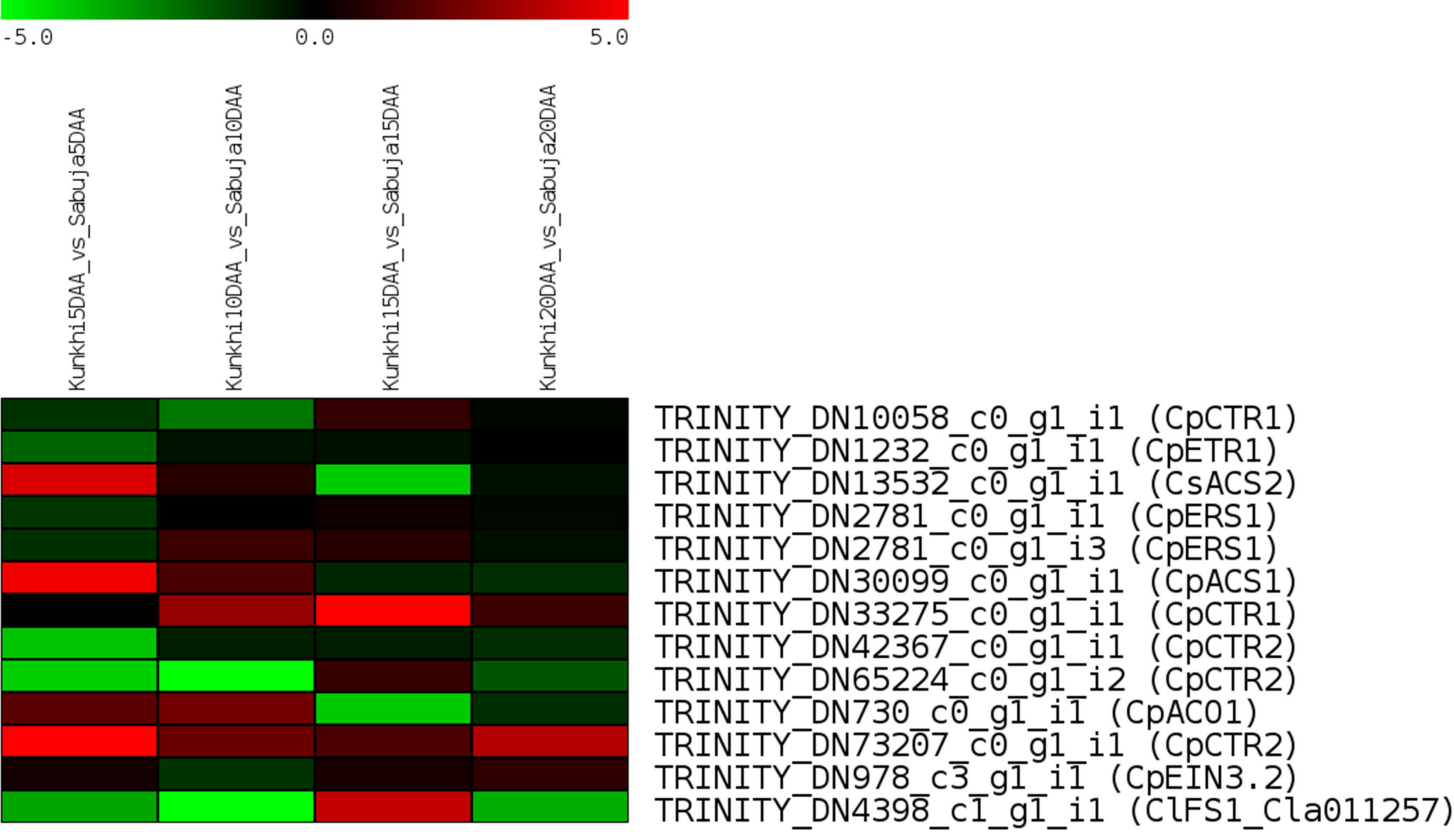
*In-silico* expression of genes reported to play crucial roles in fruit development in cucurbits. The scale represents Log_2_FC between the fruits of Kunkhi and Sabuja varieties generated using RSEM and edgeR.

In addition to analyzing the unigenes obtained in this study, we also investigated the expression patterns for genes previously reported in fruit development and morphology in cucurbits (Dou et al., 2018; Pan et al., 2020; Boualem et al., 2022) We extracted the sequences for these genes using the information provided in their respective publications and identified their homologs in the *C. grandis* transcriptome using BLASTN and subjected them to *in-silico* gene expression analyses (**Supplementary Table S4**). We observed that most of the genes related to ethylene biosynthesis such as homologs of *CpETR1, CpERS1, CpCTR2* and *CpEIN3*.2 were downregulated in ANKu as compared with ANSa. It was interesting to note that homologs of the genes *Cla011257* was downregulated in all stages of development in AnKu, except at 15 DAA while another homolog of *CpCTR1* was upregulated at 10, 15 and 20 DAA in ANKu as compared with ANSa (**Figure 7**). It was also curious to note that few of the crucial genes like *CpACS7* did not find a homolog in *C. grandis* transcriptome.

### 3.4 Gene enrichment at different stage of fruit development

Taking ANKu as a reference, we analyzed the DEUs that were enriched in each stage of fruit development of the varieties of Ivy gourd to look for molecular functions that could be involved in regulating fruit morphology in ANKu and ANSa. The results showed that various binding activities like “nucleotide binding”, “ATP binding” and catalytic activities like “hydrolase” and “transferase” activity were differentially regulated at 5 DAA. These activities were also found to be enriched at 10 DAA with the addition of “transporter” and “pyrophosphatase” activity. The later stages of 15 and 20 DAA showed a drastic change in the gene enrichment pattern with a clear reduction in the representation of catalytic activity like “transporter” (**Supplementary Figures S4A–D**).

### 3.5 Transcription factors regulating fruit development and morphology in *C. grandis*

**Figure 8:**
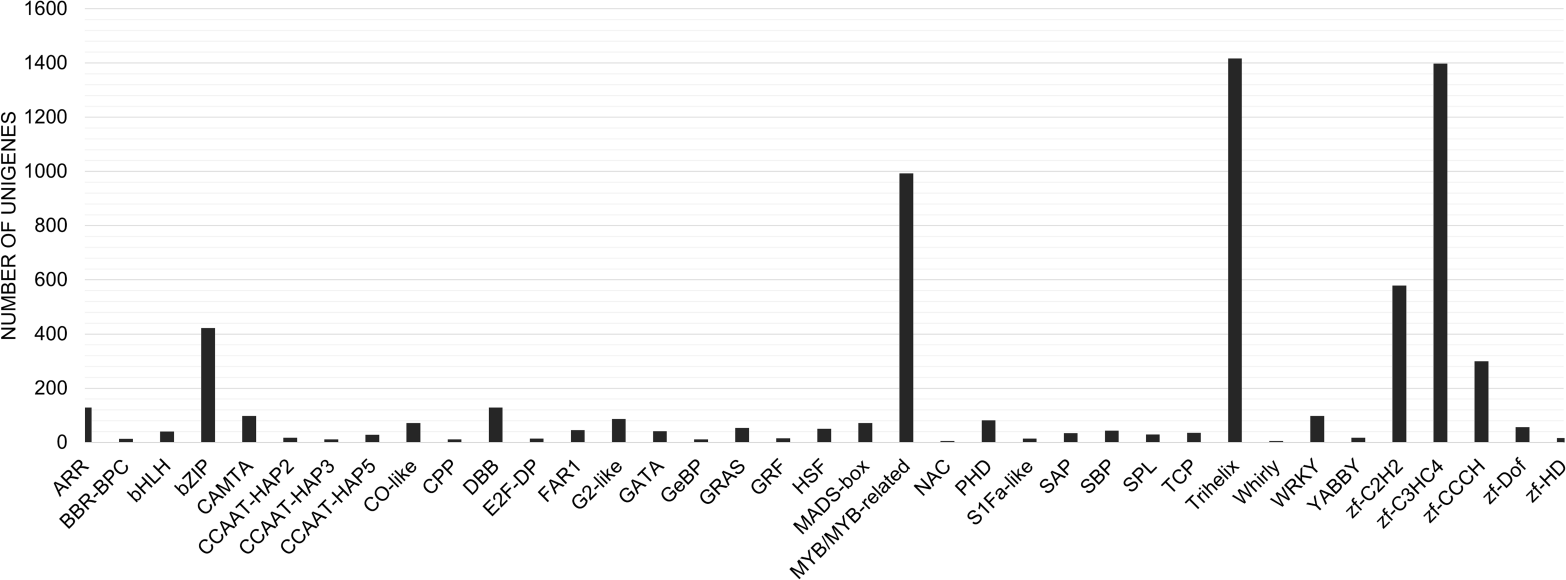
Distribution of different transcription factor families in the transcriptome of Ivy gourd. A bar graph representing Ivy gourd unigenes encoding transcription factors belonging to the most well represented families.

**Figure 9:**
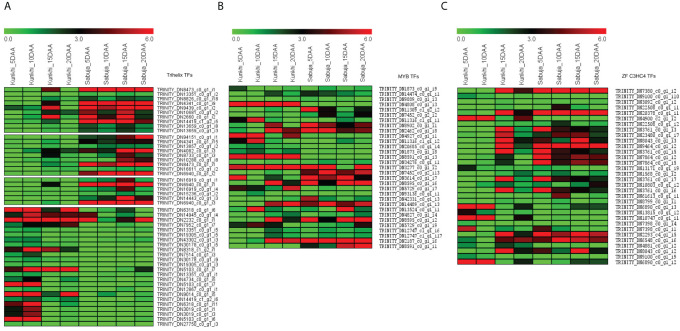
Expression profile of the most abundant transcription factors in Ivy gourd transcriptome; **(A)** Trihelix **(B)** MYB and **(C)** Zinc finger C3HC4 type. The heat map was generated using MeV software after averaging the normalized expression values obtained after edgeR.

We identified a total of 7,635 unigenes coding for some of the most widely studied transcription factors in the transcriptome of *C. grandis* using the HMM profiles of individual TF families. The Trihelix (1417), zf-C3HC4 (1398) and MYB/MYB related (993) transcription factors accounted for the majority (**Figure 8**). The expression profiles for these three families of TFs in the two varieties of *C. grandis* showed that a number of Trihelix TFs were differentially expressed in ANSa as compared to ANKu. Some of these unigenes such as TRINITY_DN8473_c0_g1_i1, TRINITY_DN4341_c0_g1_i9, TRINITY_DN14419_c1_g2_i5, TRINITY_DN94151_c0_g1_i1, TRINITY_DN8473_c0_g1_i1 and TRINITY_DN6940_c0_g1_i2 were upregulated in the ANSa variety (**Figure 9A**). On the other hand, the unigenes TRINITY_DN6318_c0_g1_i6, TRINITY_DN7952_c0_g1_i7, TRINITY_DN8318_c1_g2_i1 and TRINITY_DN5103_c0_g1_i7 were downregulated in ANSa (**Figure 9A**). Such clear preferential expression was not observed for the MYB and zf-C3HC4 TFs (**Figures 9B, C**). However, we observed that the unigenes TRINITY_DN 4808_c0_g1_i3 and TRINITY_DN8591_c0_g1_i3, coding for MYB TFs were downregulated in the ANSa variety while TRINITY_DN7452_c0_g2_i13 was upregulated in ANSa as compared with ANKu (**Figure 9B**).

### 3.6 SSRs identified in *C. grandis* transcriptome

We searched for simple sequence repeats (SSRs) in the unigenes of Ivy gourd and identified 98768 SSRs in 155205 unigenes. Majority of these SSRs were mononucleotide repeats (64914), followed by trinucleotide repeats (18702) (**Table 2**). Since we have identified these SSRs in the transcriptome of Ivy gourd, these would be regarded as genic SSRs and have a high possibility of being associated with protein coding genes. Hence, we also analyzed the SSRs in the differentially expressed subset of unigenes and found that a number of these SSRs were present in the differentially expressed unigenes which were annotated to code for various transcription factors, genes involved in biosynthesis of metabolites, ubiquitin mediated proteolysis, structural elements and signalling pathways (**Supplementary Table S6**).

**Table 2:** Details of the SSRs identified in the transcriptome of Ivy gourd.

Total number of sequences examined	155205
Total size of examined sequences (bp)	127048296
Total number of identified SSRs	98768
Number of SSR containing sequences	59776
Number of sequences containing more than 1 SSR	23815
Number of SSRs present in compound formation	16919
Distribution to different repeat type classes	
Mono-nucleotide repeats	64914
Di- nucleotide repeats	5122
Tri-nucleotide repeats	18702
Tetra-nucleotide repeats	2354
Penta-nucleotide repeats	4489
Hexa-nucleotide repeats	3187

We designed primers for 20 SSRs associated with DEUs and were able to successfully amplify 16 through PCR in three biological replicates of 15DAA fruit tissue (**Supplementary Figure S5**). These SSRs were associated with unigenes coding for Leucine-rich repeat extensin-like protein, Trihelix transcription factor and 3-hydroxybutyryl-CoA dehydrogenase (**Supplementary Table S7**). The initial observations suggest that few of the SSRs, such as CgSSRs 10, 11 and 14, could be polymorphic (**Supplementary Figure S5**).

### 3.7 qRT- PCR based gene expression analysis

**Figure 10:**
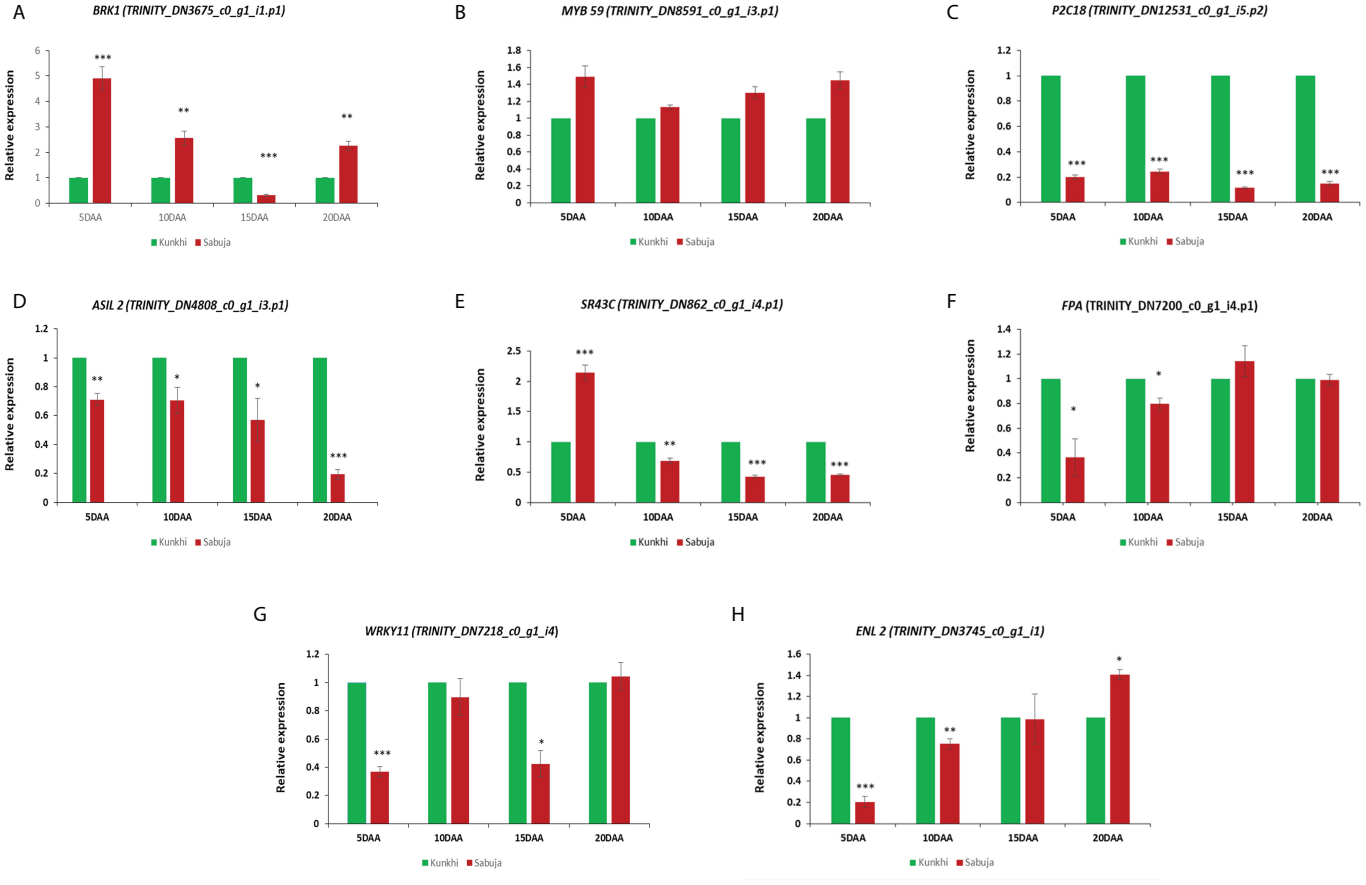
Expression analysis of selected DEUs **(A–H)** from fruit tissue of both the varieties ANKu and ANSa by qRT- PCR using *18S rRNA* as reference gene. Values with asterisk (*) are significantly different from other variety at *p ≤*0.05, as analyzed by unpaired t test.

We performed quantitative real time PCR assay to assess the correlation between the *in silico* expression data and qRT- PCR based expression analysis. The analysis suggests a good correlation between the two sets of data (**Supplementary Table S8**) where we used leaf tissue samples as the reference. We then performed qRT-PCR analysis for 8 genes annotated as *MYB59* (TRINITY_DN8591_c0_g1_i3.p1), *P2C18* (TRINITY_DN12531_c0_g1_i5.p2), *SR43C* (TRINITY_DN862_c0_g1_i4.p1), *FPA* (TRINITY_DN7200_c0_g1_i4.p1), *BRK1* (TRINITY_DN3675_c0_g1_i1.p1), *ASIL2* (TRINITY_DN4808_c0_g1_i3.p1), *WRKY11* (TRINITY_DN7218_c0_g1_i4), *ENL* 2 (TRINITY_DN3745_c0_g1_i1) to observe their expression in the two varieties of Ivy gourd taking ANKu as reference and compared with those of ANSa to make a comparative expression analysis between the developing fruits of the two varieties (**Figure 10**). The qRT PCR analysis showed significant upregulation of *BRK-1* gene in all the developmental stages of ANSa as compared with ANKu, with maximum upregulation of *BRK*-*1* in 5 DAA stage of ANSa. The transcription factor *MYB 59* showed slight upregulation in all developmental stages of ANSa. Other genes such as *P2C18*, *ASIL 2* were found to be downregulated in all the stages of development in ANSa. *SR43C* showed upregulation in only in the 5DAA tissue sample in ANSa while *ENL2* was upregulated in 20DAA tissue. Two genes *FPA* and *WRKY11* were generally down regulated or showed no significant change in expression in ANSa.

## 4 Discussion

Cucurbitaceae is the second largest family bearing diverse groups of plant with broad range of fruit characteristic (Shaina and Suhara Beevy, 2012; Ma et al., 2020). Studies related to fruit development and variations in fruit sizes are also reported in other plants like Arabidopsis, tomato, pear, pumpkin, cucumber, bottle gourd, apple, Apricot, sweet cherry among many others (Jiang et al., 2015; Xanthopoulou et al., 2017; Di Marzo et al., 2020; Yuste-Lisbona et al., 2020; Zhang et al., 2020; Kuhn et al., 2021; Liu et al., 2023; Wang et al., 2022; Jahed and Hirst, 2023). RNA-Seq analyses have become the most popular method to identify the key genes regulating fruit morphology in several cucurbit vegetables (Zhang et al., 2016; Ma et al., 2020; Chen et al., 2021). Ivy gourd is a wonderful vegetable for its nutritive as well as medicinal value. However, research in this crop is very limited, and genomic information is scarce. Here, we attempt to address the patterns of gene expression that influence fruit size and factors that are heavily influenced by the process of cell division and cell expansion.

### 4.1 Anatomical characterization reveals the role of cell numbers and size in fruit size variation between two varieties of *C. grandis*

Generally, during development, a fruit undergoes significant growth in size due to cell divisions and cell enlargement that determine fruit size. Interestingly, in the present study, we found a similar pattern of growth in cell numbers during fruit developmental stages of both the ANKu and ANSa varieties. The number of cells in fruits of each variety start increasing from 5 DAA to 15 DAA and then stagnate at maturity. This pattern agrees with the normal growth pattern of fruit where more cell division takes place at the early stages of fruit development that determines its fruit size and at maturity cell division slows down or stops to check further growth (Yang et al., 2013). However, the number of cells was found to be higher in ANKu fruits than in ANSa. Reduced cell number are known to result in smaller fruit size in cucumber (Yang et al., 2018) and some previous histological studies also suggest the role of cell number in fruit size determination of melon (Higashi et al., 1999), sweet cherry (Olmstead et al., 2007), peach (Scorzal et al., 1991), pear (Zhang et al., 2007). Therefore, the present study suggests that the longer fruits of ANKu are a result of a higher cell number. Apart from cell number, cell area or expansion also plays an important role in fruit size or shape determination (Liu et al., 2020). In the present study, greater cell area was observed in ANSa compared to ANKu. Maximum expansion was found in 15 DAA and 20 DAA fruit of ANSa and ANKu as compared to earlier stages of development, suggesting that cells expand during mature stages of fruit development before ripening. Such observations lead us to believe that greater cell area and lower cell number in ANSa makes the fruit short and oval or conical while the fruits of ANKu may be long and slender due to larger number of cells and less expansion. Previous studies on apple and pineapple also report fruit size determination through cell number and expansion (Harada et al., 2005; Li et al., 2010). These observations act as a guide to look for the molecular bases of these specific processes for a better understanding of fruit development in Ivy gourd.

### 4.2 Fruit shape and size in *C. grandis* may be a result of varied gene regulatory networks

So far, we understood that, variation in cell number leads to fruit size variations and that cell number is influenced by cell division. Various genetic factors, hormones can influence the rate of cell division and knowing those factors could help us to identify fruit size regulators. We have incorporated gene expression analysis to identify DEUs that may regulate fruit size in Ivy gourd. Cell division and cell expansion is often associated with reorganization of structural elements such as microtubules and actins (Gibieža and Petrikaitė, 2021). It has been reported that *BRICK 1 or BRK 1* gene plays an important role in promoting actin polymerization (Djakovic et al., 2006). In our study, *BRK1* was up regulated in 5 DAA fruits and 20 DAA fruits of ANSa as compared to the respective stages of ANKu, emphasizing the role of this gene in fruit development. Higher expression of *BRK1* in early stage of fruit development in ANSa might have role in extending its width and down-regulation in ANKu probably helps in reducing the width and focusing on increase in length. The higher expression of *BRK1* in earlier stages of fruit development also aligns with studies that suggest that the fruit size is determined in early stages of its development (Pan et al., 2020). Transcription factors like *MYB* are known to be involved in numerous roles in fruit development, quality, ripening etc (Wang et al., 2023; Zhang et al., 2024). *MYB* is involved in auxin biosynthesis and regulates fruit size in pear and citrus fruits (Wang et al., 2022; Zhang et al., 2023). *MYB 59* belongs to the *MYB* transcription factor family that promotes cell growth in vegetative parts of plants including roots and shoots (Fasani et al., 2019). In this study, *MYB59* showed negligible change in expression between the ANKu and ANSa varieties suggesting that the gene may be involved in processes of fruit development that do not relate to fruit size in Ivy gourd. Protein phosphatases are a group of proteins that have not been extensively studied in plants, however, fruit development and ripening can be regulated by protein phosphatase 2C by some unknown mechanism (Liang et al., 2021). Previous reports also suggest that protein phosphatase negatively controls ethylene biosynthesis (protein phosphatase deficient plants synthesize more ethylene) by regulating the enzyme 1-aminocyclopropane 1-carboxylate synthase (ACS) (Park et al., 2021) and ethylene is a negative regulator of cell expansion in most of the tissues (Skottke et al., 2011). In the present study, we have examined the expression of *P2C18* gene, which encodes probable protein phosphatase 2C, to be lower in ANSa which suggests that, *P2C18* downregulation may promote high ethylene production in fruit tissue of ANSa that lowers the vertical expansion of fruit axis, making the fruit shorter in length. In other words, upregulation of *P2C18* in ANKu may cause low ethylene production thereby supporting higher cell expansion of fruit axis that makes the fruit elongated. *ASIL2* is a transcription factors belonging to the trihelix DNA binding protein family. Role of *ASIL* in fruit development is not yet reported, however it has been reported to have role in seed maturation and vegetative growth (Hussain et al., 2020; Ruiz et al., 2021). In the present study, *ASIL* 2 is downregulated in all the developmental stages of ANSa, and therefore, can be considered to have higher expression in ANKu, which suggests its role in elongation of fruit by some unknown mechanism. In addition to this, we also observed expression of gene *SR43C* which codes for chloroplast signal recognition particle and has role in chlorophyll synthesis and fruit quality (Huo et al., 2023). Higher expression of *SR43C* in early stage of fruit development (5 DAA) in ANSa may imply a potential role of *SR43C* gene in determining fruit size through some unknown mechanisms. Early stage of fruit development is dependent on flowering time, which is regulated by flower time control protein or *FPA*. It is a nuclear protein that regulates flowering by silencing the mRNA encoding the repressor protein *FLC* (Hornyik et al., 2010). In the present study, *FPA* has similar expression in ANSa as compared with ANKu which suggests that the fruit morphology is unaffected by the *FPA* gene. The *WRKY* gene family is one of the important families of transcription factors, known to have a role in plant stress and various plant physiological processes, however, no reports have been found about the involvement of *WRKY* in fruit size regulation (Ali et al., 2018). However, the members of the *WRKY* TF family are reported to be involved in fruit ripening and enlargement (Liu et al., 2023). In the present study, the *WRKY 11* was found to be generally down-regulated in ANSa which suggests that the homolog of *WRKY11* in Ivy gourd could have a role in fruit elongation. The expressions of *ENL*2 gene that encodes early nodulin like protein 2, was also examined in fruit tissues of both the verities. The early nodulin like proteins play major role in transport of nutrients, solutes, amino acids and hormones in plants (Denance et al., 2014). A previous study of watermelon, reported that, downregulation of nodulin like protein 2 inhibits fruit expansion (Gao et al., 2023). In the present study, *ENL2*, was found to be downregulated in all the stages of fruit development of ANSa except the matured stage (20 DAA). This result can conclude that, fruit expansion is inhibited or slow during early stages of fruit development that makes the fruit short and sudden up-regulation in matured stage may contribute to its lateral expansion making the fruit conical or oval.

There are reports in cucurbits that indicate the process of ethylene biosynthesis is inversely co-related to fruit elongation (Martínez et al., 2013). In this study, we found the homologs of some of the members of ethylene biosynthesis pathway to be down-regulated in the elongated ANKu fruits as compared with the shorter ANSa. It was also interesting to note that another well characterized gene studied in watermelon, *Cla011257* (Pan et al., 2020), was down-regulated in all stages of ANKu except in 15 DAA. This could be a discrepancy related to the completeness of the transcriptome and warrants further investigation. The next step would be to generate a more comprehensive genomic map of *C.grandis* to include non-coding elements and complete gene sequences. Such studies have been undertaken in *C.grandis* where the authors have studied the evolution of Y chromosome (Janousek et al., 2022). Similar efforts need to be made to add to the genomic resources of *C.grandis* to facilitate future efforts in plant improvement. However, all the above hypotheses are based on the data provided through RNA-seq and molecular characterization using quantitative PCR analyses. These genes need to be characterized for their functional roles in plant system using tools for gene manipulation which have been developed recently for cucurbits (Devani et al., 2020) and can be taken up in future to report a more definitive role of these genes in fruit development in an economically important crop like *C. grandis*. However, the transformation of cucurbits with target genes is still in its infancy and will require considerable standardizations for Ivy gourd. Therefore, a more feasible approach would be to identify molecular markers associated with the traits of interest and use them for selective breeding.

### 4.3 Transcription factors may be implicated in fruit development and morphology regulation

Transcription factors (TFs) are a varied group of DNA binding protein that interact with cis-acting elements in promoter sequence of target genes thereby regulating gene expression (Cheng et al., 2019). In this study, we have observed that the Trihelix transcription factor (*TTF*) gene family, also referred to as the GT transcription factor family, is differentially expressed in ANKu and ANSa. The members of this family have been reported to play important roles in overall plant developmental process (Cheng et al., 2019). The *ASIL1* gene, a member of TTF DNA binding protein family, regulates silique size in *Arabidopsis* (Gao et al., 2009). Another TF family that was conspicuous in this study is the *MYB* transcription factor family, which primarily function in cell cycle regulation, particularly in the G2/M phase (Kranz et al., 2000; Stracke et al., 2001). C3HC4-type RING finger proteins represent one of the largest transcription factors known for various plant processes like regulation of growth and development, signalling network and abiotic stress and fruit development (Wu et al., 2014; Agarwal and Khurana, 2020). Although genes encoding *TTF*, *MYB* and *C3HC4*-type *RING* finger containing proteins were most abundant in the Ivy gourd transcriptome, given that the unigenes encoding Trihelix TFs showed better differential expression compared with *MYB* and *C3HC4*-type *RING* finger proteins, they could be good candidates for further characterization and it would be interesting to identify molecular markers associated with these genes.

### 4.4 SSR markers can be developed as a valuable resource for marker assisted selection

DNA markers play crucial role in plant breeding programmes to develop new superior varieties with desirable traits and simple sequence repeats (SSRs) or microsatellites markers are one of the most widely studied groups given that maximum mutation rates are observed in SSRs making them a crucial component of genome evolution (Kashi et al., 1997). SSRs are desirable molecular markers with several applications such as determination of functional genetic variations such as paternity determination, genetic diversity assessment, and population genetics studies and for the development of a genetic map (Xanthopoulou et al., 2017). Nowadays transcriptome sequencing is highly evolved that provides a rapid, cost effective and reliable source of expression datasets in non-model species and also facilitates the development of SSRs using bioinformatics tools (Marioni et al., 2008; Zhang et al., 2012). Transcriptomes are a good source of genic SSRs which are linked with the loci that mostly focus on morphology (Lal et al., 2017). In comparison with genomic SSR markers, the genic SSR markers may facilitate the identification of candidate functional genes and could expand the efficiency of marker assisted selection (Gupta and Rustgi, 2004; Zhang et al., 2012). In Ivy gourd, presence of SSRs in the differentially expressed unigenes coding for transcription factors, biosynthesis of metabolites, ubiquitin mediated proteolysis, structural element and signalling pathways which are crucial in fleshy fruit development and ripening (Jia et al., 2023; Wang et al., 2023), indicates their potential for developing functional molecular markers. Our initial assessment also indicates that few of the SSRs are polymorphic in nature, and associated with genes encoding Leucine-rich repeat extensin-like protein, Trihelix transcription factor and 3-hydroxybutyryl-CoA dehydrogenase, implying that analysis and subsequent sequencing efforts at larger scales will help identify numerous polymorphic SSRs in *C.grandis* that can be used for marker assisted selection for crop breeding as well as for diversity analysis in plants (Kapoor et al., 2023; Li et al., 2023).

## 5 Conclusion

This study reports the transcriptome of Ivy gourd (*Coccinia grandis)*, an important and popular food crop of India, and attempts to explore the differences in patterns of gene expression in two cultivated varieties of the plant with considerably different fruit morphologies. It showcases one of the most widely utilized functions of the modern-day “omics” platforms that allow understanding the molecular basis of important biological processes of an organism. This study involves various stages of fruit development and has led to the identification of genes that may have a crucial role in fruit development as well as in determining fruit size and shape. This study has also generated a variety of genomic resources for Ivy gourd in form of genic SSRs which will provide new avenues for research into fruit morphology in Cucurbitaceae family. These resources will not only enrich the existing repertoire of molecules available for cucurbits, but also shed light on a crop like Ivy gourd, which has great economic potential. Polymorphic molecular markers will prove to be an especially valuable genomic resource for the crop and facilitate future breeding attempts for improving crop quality. Overall, this study provides a foundation for further exploration of molecular basis of fruit size regulation in *C. grandis* varieties.

## Data availability statement

The datasets presented in this study can be found in online repositories. The names of the repository/repositories and accession number(s) can be found below: Bioproject accession number: PRJNA851184.

## Ethics statement

We confirm that the experimental research and field studies was in accordance with relevant institutional, national, and international guidelines and legislation.

## Author contributions

MP: Writing – original draft, Validation, Investigation, Data curation. SP: Writing – review & editing, Writing – original draft, Methodology, Investigation, Formal analysis, Data curation, Conceptualization. PM: Writing – review & editing, Supervision, Investigation, Formal analysis, Conceptualization.

## References

[B1] Abbas H. M. K. Huang H.-X. Wang A.-J. Wu T.-Q. Xue S.-D. Ahmad A.. (2020). Metabolic and transcriptomic analysis of two Cucurbita moschata germplasms throughout fruit development. BMC Genomics 21, 1–13. doi: 10.1186/s12864-020-6774-y PMC7227267 32414328

[B2] Agarwal P. Khurana P. (2020). TaZnF, a C3HC4 type RING zinc finger protein from Triticum aestivum is involved in dehydration and salinity stress. J. Plant Biochem. Biotechnol. 29, 395–406. doi: 10.1007/s13562-019-00546-8

[B3] Ali M. A. Azeem F. Nawaz M. A. Acet T. Abbas A. Imran Q. M.. (2018). Transcription factors WRKY11 and WRKY17 are involved in abiotic stress responses in arabidopsis. J. Plant Physiol. 226, 12–21. 29689430 10.1016/j.jplph.2018.04.007

[B4] Azzi L. Deluche C. Gévaudant Frédéric Frangne N. Delmas F. Hernould M.. (2015). Fruit growth-related genes in tomato. J. Exp. Bot. 66, 1075–1086. doi: 10.1093/jxb/eru527 25573859

[B5] Beier S. Thiel T. Münch T. Scholz U. Mascher M. (2017). MISA-web: a web server for microsatellite prediction. Bioinformatics 33, 2583–2585. doi: 10.1093/bioinformatics/btx198 28398459 PMC5870701

[B6] Boualem A. Berthet S. Devani R. S. Camps C. Fleurier S. Morin H.. (2022). Ethylene plays a dual role in sex determination and fruit shape in cucurbits. Curr. Biol. 32, 2390–2401. doi: 10.1016/j.cub.2022.04.031 35525245

[B7] Chen S. Li Y. Zhao Y. Li G. Zhang W. Wu Y.. (2021). iTRAQ and RNA-Seq analyses revealed the effects of grafting on fruit development and ripening of oriental melon (Cucumis melo L. var. makuwa). Gene 766, 145142. doi: 10.1016/j.gene.2020.145142 32911027

[B8] Cheng X. Xiong R. Yan H. Gao Y. Liu H. Wu M.. (2019). The trihelix family of transcription factors: functional and evolutionary analysis in Moso bamboo (Phyllostachys edulis). BMC Plant Biol. 19, 1–20. doi: 10.1186/s12870-019-1744-8 31023225 PMC6482567

[B9] Chevalier C. Bourdon M. Pirrello J. Cheniclet C. Gévaudant F. Frangne N. (2014). Endoreduplication and fruit growth in tomato: evidence in favour of the karyoplasmic ratio theory. J. Exp. Bot. 65, 2731–2746. doi: 10.1093/jxb/ert366 24187421

[B10] Denancé N. Szurek B. Noël L. D. (2014). Emerging functions of nodulin-like proteins in non-nodulating plant species. Plant Cell Physiol. 55 (3), 469–474. 24470637 10.1093/pcp/pct198

[B11] Devani R. S. Kute A. John S. Adhikari S. Sinha S. Banerjee A. K. (2020). Development of a virus-induced gene silencing system for dioecious coccinia grandis. Mol. Biotechnol. 62, 412–422. doi: 10.1007/s12033-020-00259-7 32592122

[B12] Di Marzo M. Herrera-Ubaldo H. Caporali E. Novák O. Strnad M. Balanzà V.. (2020). SEEDSTICK controls Arabidopsis fruit size by regulating cytokinin levels and FRUITFULL. Cell Rep. 30, 2846–2857. doi: 10.1016/j.celrep.2020.01.101 32101756

[B13] Djakovic S. Dyachok J. Burke M. Frank M. J. Smith L. G. (2006). BRICK1/HSPC300 functions with SCAR and the ARP2/3 complex to regulate epidermal cell shape in Arabidopsis. Development. 133, 1091–1100. doi: 10.1242/dev.02280 16481352

[B14] Dou J. Zhao S. Lu X. He N. Zhang L. Ali A.. (2018). Genetic mapping reveals a candidate gene (ClFS1) for fruit shape in watermelon (Citrullus lanatus L.). Theor. Appl. Genet. 131, 947–958. doi: 10.1007/s00122-018-3050-5 29362832

[B15] Fasani E. DalCorso G. Costa A. Zenoni S. Furini A. (2019). The Arabidopsis thaliana transcription factor MYB59 regulates calcium signalling during plant growth and stress response. Plant Mol. Biol. 99, 517–534. doi: 10.1007/s11103-019-00833-x 30710226

[B16] Gao M.-J. Lydiate D. J. Li X. Lui H. Gjetvaj B. Hegedus D. D.. (2009). Repression of seed maturation genes by a trihelix transcriptional repressor in Arabidopsis seedlings. Plant Cell 21, 54–71. doi: 10.1105/tpc.108.061309 19155348 PMC2648069

[B17] Gao W. She F. Sun Y. Han B. Wang X. Xu G. (2023). Transcriptome analysis reveals the genes related to water-melon fruit expansion under low-light stress. Plants 12 (4), 935. 36840282 10.3390/plants12040935 PMC9958833

[B18] Gibieža P. Petrikaitė V. (2021). The regulation of actin dynamics during cell division and Malignancy. Am. J. Cancer Res. 11, 4050. 34659876 PMC8493394

[B19] Gillaspy G. Ben-David H. Gruissem W. (1993). Fruits: a developmental perspective. Plant Cell 5, 1439. doi: 10.1105/tpc.5.10.1439 12271039 PMC160374

[B20] Guo M. Rupe M. A. Dieter Jo A. Zou J. Spielbauer D. Duncan K. E.. (2010). Cell Number Regulator1 affects plant and organ size in maize: implications for crop yield enhancement and heterosis. Plant Cell 22, 1057–1073. doi: 10.1105/tpc.109.073676 20400678 PMC2879740

[B21] Gupta P. K. Rustgi S. (2004). Molecular markers from the transcribed/expressed region of the genome in higher plants. Funct. Integr. Genomics 4, 139–1625. doi: 10.1007/s10142-004-0107-0 15095058

[B22] Haas B. J. Papanicolaou A. Yassour M. Grabherr M. Blood P. D. Bowden J.. (2013). *De novo* transcript sequence reconstruction from RNA-seq using the Trinity platform for reference generation and analysis. Nat. Protoc. 8, 1494–1512. doi: 10.1038/nprot.2013.084 23845962 PMC3875132

[B23] Harada T. Kurahashi W. Yanai M. Wakasa Y. Satoh T. (2005). Involvement of cell proliferation and cell enlargement in increasing the fruit size of Malus species. Scientia Hortic. 105, 447–456. doi: 10.1016/j.scienta.2005.02.006

[B24] Higashi K. Hosoya K. Ezura H. (1999). Histological analysis of fruit development between two melon (Cucumis melo L. reticulatus) genotypes setting a different size of fruit. J. Exp. Bot. 50, 1593–1597. doi: 10.1093/jxb/50.339.1593

[B25] Hornyik C. Terzi L. C. Simpson G. G. (2010). The spen family protein FPA controls alternative cleavage and polyadenylation of RNA. Dev. Cell 18, 203–213. doi: 10.1016/j.devcel.2009.12.009 20079695

[B26] Huang M. Zhu X. Bai H. Wang C. Gou N. Zhang Y.. (2023). Comparative anatomical and transcriptomics reveal the larger cell size as a major contributor to larger fruit size in apricot. Int. J. Mol. Sci. 24, 8748. doi: 10.3390/ijms24108748 37240096 PMC10218707

[B27] Huo Y. Zhang G. Wang P. (2023). Forward genetic studies reveal LsAPRR2 as a key gene in regulating the green color of pericarp in bottle gourd (Lagenaria siceraria). Front. Plant Sci. 14, 1130669. doi: 10.3389/fpls.2023.1130669 36875578 PMC9975725

[B28] Hussain Q. Shi J. Scheben A. Zhan J. Wang X. Liu G.. (2020). Genetic and signalling pathways of dry fruit size: targets for genome editing-based crop improvement. Plant Biotechnol. J. 18, 1124–1140. doi: 10.1111/pbi.13318 31850661 PMC7152616

[B29] Jahed K. R. Hirst P. M. (2023). Fruit growth and development in apple: a molecular, genomics and epigenetics perspective. Front. Plant Sci. 14, 1122397. doi: 10.3389/fpls.2023.1122397 37123845 PMC10130390

[B30] Janousek B. Gogela R. Bacovsky V. Renner S. S. (2022). The evolution of huge Y chromosomes in Coccinia grandis and its sister, Coccinia schimperi. Philos. Trans. R. Soc. B 377, 20210294. doi: 10.1098/rstb.2021.0294 PMC8935295 35306898

[B31] Jia W. Liu G. Zhang P. Li H. Peng Z. Wang Y.. (2023). The ubiquitin–26S proteasome pathway and its role in the ripening of fleshy fruits. Int. J. Mol. Sci. 24, 2750. doi: 10.3390/ijms24032750 36769071 PMC9917055

[B32] Jiang L. Yan S. Yang W. Li Y. Xia M. Chen Z.. (2015). Transcriptomic analysis reveals the roles of microtubule-related genes and transcription factors in fruit length regulation in cucumber (Cucumis sativus L.). Sci. Rep. 5, 8031. doi: 10.1038/srep08031 25619948 PMC5379036

[B33] Kapoor B. Sharma M. Sharma R. Zadokar A. Thakur A. Sharma P.. (2023). *De novo* transcriptome profiling and development of novel secondary metabolites based genic SSRs in medicinal plant Phyllanthus emblica L.(Aonla). Sci. Rep. 13, 17319. doi: 10.1038/s41598-023-44317-x 37828031 PMC10570353

[B34] Kashi Y. King D. Soller M. (1997). Simple sequence repeats as a source of quantitative genetic variation. Trends Genet. 13, 74–78. doi: 10.1016/S0168-9525(97)01008-1 9055609

[B35] Kranz H. Scholz K. Weisshaar B. (2000). c-MYB oncogene-like genes encoding three MYB repeats occur in all major plant lineages. Plant J. 21, 231–235. doi: 10.1046/j.1365-313x.2000.00666.x 10743663

[B36] Kuhn N. Maldonado J. Ponce C. Arellano M. Time A. Multari S.. (2021). RNAseq reveals different transcriptomic responses to GA3 in early and midseason varieties before ripening initiation in sweet cherry fruits. Sci. Rep. 11, 13075. doi: 10.1038/s41598-021-92080-8 34158527 PMC8219793

[B37] Lal S. Singh A. K. Singh S. K. Srivastav M. Singh B. P. Sharma N.. (2017). Association analysis for pomological traits in mango (Mangifera indica L.) by genic-SSR markers. Trees 31, 1391–1409. doi: 10.1007/s00468-017-1554-2

[B38] Langmead B. Salzberg S. L. (2012). Fast gapped-read alignment with Bowtie 2. Nat. Methods 9, 357–359. doi: 10.1038/nmeth.1923 22388286 PMC3322381

[B39] Li W. Godzik A. (2006). Cd-hit: a fast program for clustering and comparing large sets of protein or nucleotide sequences. Bioinformatics 22, 1658–1659. doi: 10.1093/bioinformatics/btl158 16731699

[B40] Li J.-W. Li H. Liu Z.-W. Wang Y.-X. Chen Y. Yang N.. (2023). Molecular markers in tea plant (Camellia sinensis): Applications to evolution, genetic identification, and molecular breeding. Plant Physiol. Biochem. 198, 107704. doi: 10.1016/j.plaphy.2023.107704 37086694

[B41] Li Y.-H. Zhang Z. Sun G.-M. (2010). Changes in cell number and cell size during pineapple (Ananas comosus L.) fruit development and their relationship with fruit size. Aust. J. Bot. 58, 673–678. doi: 10.1071/BT10225

[B42] Liang B. Sun Y. Wang J. Zheng Y. Zhang W. Xu Y.. (2021). Tomato protein phosphatase 2C influences the onset of fruit ripening and fruit glossiness. J. Exp. Bot. 72, 2403–2418. doi: 10.1093/jxb/eraa593 33345282

[B43] Libault M. Zhang X.-C. Govindarajulu M. Qiu J. Ong Y. T. Brechenmacher L.. (2010). A member of the highly conserved FWL (tomato FW2. 2-like) gene family is essential for soybean nodule organogenesis. Plant J. 62, 852–864. doi: 10.1111/tpj.2010.62.issue-5 20230508

[B44] Liu X. Pan Y. Liu C. Ding Y. Wang X. Cheng Z.. (2020). Cucumber fruit size and shape variations explored from the aspects of morphology, histology, and endogenous hormones. Plants 9, 772. doi: 10.3390/plants9060772 32575654 PMC7356835

[B45] Liu H. Zhang X. Li J. Zhang G. Fang H. Li Y. (2023). Transcriptome analysis reveals the mechanism of different fruit appearance between apricot (Armeniaca vulgaris lam.) and its seedling. Mol. Biol. Rep. 50 (10), 7995–8003. 37540452 10.1007/s11033-023-08631-x

[B46] Livak K. J. Schmittgen T. D. (2001). Analysis of relative gene expression data using real-time quantitative PCR and the 2– ΔΔCT method. methods 25, 402–408. doi: 10.1006/meth.2001.1262 11846609

[B47] Luo W. Li Y. Sun Y. Lu L. Zhao Z. Zhou J.. (2021). Comparative RNA-seq analysis reveals candidate genes associated with fruit set in pumpkin. Scientia Hortic. 288, 110255. doi: 10.1016/j.scienta.2021.110255

[B48] Ma L. Wang Q. Mu J. Fu A. Wen C. Zhao X.. (2020). The genome and transcriptome analysis of snake gourd provide insights into its evolution and fruit development and ripening. Horticulture Res. 7. doi: 10.1038/s41438-020-00423-9 PMC7704671 33328440

[B49] Marioni J. C. Mason C. E. Mane S. M. Stephens M. Gilad Y. (2008). RNA-seq: an assessment of technical reproducibility and comparison with gene expression arrays. Genome Res. 18, 1509–1517. doi: 10.1101/gr.079558.108 18550803 PMC2527709

[B50] Martínez C. Manzano S. Megías Z. Garrido D. Picó B. Jamilena M. (2013). Involvement of ethylene biosynthesis and signalling in fruit set and early fruit development in zucchini squash (Cucurbita pepo L.). BMC Plant Biol. 13, 1–14. doi: 10.1186/1471-2229-13-139 24053311 PMC3856489

[B51] Mauxion J.-P. Chevalier C. Gonzalez N. (2021). Complex cellular and molecular events determining fruit size. Trends Plant Sci. 26, 1023–1038. doi: 10.1016/j.tplants.2021.05.008 34158228

[B52] Mohanty J. N. Nayak S. Jha S. Joshi R. K. (2017). Transcriptome profiling of the floral buds and discovery of genes related to sex-differentiation in the dioecious cucurbit Coccinia grandis (L.) Voigt. Gene 626, 395–406. doi: 10.1016/j.gene.2017.05.058 28578021

[B53] Mu R.-L. Cao Y.-R. Liu Y.-F. Lei G. Zou H.-F. Liao Y.. (2009). An R2R3-type transcription factor gene AtMYB59 regulates root growth and cell cycle progression in Arabidopsis. Cell Res. 19, 1291–1304. doi: 10.1038/cr.2009.83 19581938

[B54] Okello R. C. O. Heuvelink E. P. de Visser P. H. B. Struik P. C. Marcelis L. F. M. (2015). What drives fruit growth? Funct. Plant Biol. 42, 817–827. doi: 10.1071/FP15060 32480724

[B55] Olmstead J. W. Iezzoni A. F. Whiting M. D. (2007). Genotypic differences in sweet cherry fruit size are primarily a function of cell number. J. Am. Soc. Hortic. Sci. 132, 697–703. doi: 10.21273/JASHS.132.5.697

[B56] Pan Y. Wang Y. McGregor C. Liu S. Luan F. Gao M.. (2020). Genetic architecture of fruit size and shape variation in cucurbits: a comparative perspective. Theor. Appl. Genet. 133, 1–21. doi: 10.1007/s00122-019-03481-3 31768603

[B57] Park C. Lee H. Y. Yoon G. M. (2021). The regulation of ACC synthase protein turnover: a rapid route for modulating plant development and stress responses. Curr. Opin. Plant Biol. 63, 102046. doi: 10.1016/j.pbi.2021.102046 33965697

[B58] Parrish N. Hormozdiari F. Eskin E. (2011). Assembly of non-unique insertion content using next-generation sequencing. BMC Bioinf. 12, S3. doi: 10.1186/1471-2105-12-S6-S3 PMC3194191 21989261

[B59] Peng Z. Zhao C. Li S. Guo Y. Xu H. Hu G.. (2022). Integration of genomics, transcriptomics and metabolomics identifies candidate loci underlying fruit weight in loquat. Horticulture Res. 9, uhac037. doi: 10.1093/hr/uhac037 PMC9071381 35137085

[B60] Robinson M. D. McCarthy D. J. Smyth G. K. (2010). edgeR: a Bioconductor package for differential expression analysis of digital gene expression data. bioinformatics 26, 139–140. doi: 10.1093/bioinformatics/btp616 19910308 PMC2796818

[B61] Ruiz K. A. Pelletier J. M. Wang Y. Feng M. J. Behr J. S. Ðào T. Q.. (2021). A reevaluation of the role of the ASIL trihelix transcription factors as repressors of the seed maturation program. Plant Direct 5 (10), e345. 34622120 10.1002/pld3.345 PMC8483069

[B62] Scorzal R. May L. G. Purnell B. Upchurch B. (1991). Differences in number and area of mesocarp cells between small-and large-fruited peach cultivars. J. Am. Soc. Hortic. Sci. 116, 861–864. doi: 10.21273/JASHS.116.5.861

[B63] Skottke K. R. Yoon G. M. Kieber J. J. DeLong A. (2011). Protein phosphatase 2A controls ethylene biosynthesis by differentially regulating the turnover of ACC synthase isoforms. PloS Genet. 7 (4), e1001370. 21533019 10.1371/journal.pgen.1001370 PMC3080859

[B64] Seymour G. B. Ryder C. D. Cevik V. Hammond J. P. Popovich A. King G. J.. (2011). A SEPALLATA gene is involved in the development and ripening of strawberry (Fragaria× ananassa Duch.) fruit, a non-climacteric tissue. J. Exp. Bot. 62, 1179–1188. doi: 10.1093/jxb/erq360 21115665 PMC3022409

[B65] Shaina T. J. Suhara Beevy S. (2012). Morphological variation and evolutionary significance of Coccinia grandis (L.) Voigt: an under-exploited cucurbitaceous vegetable crop. Plant systematics Evol. 298, 653–6595. doi: 10.1007/s00606-011-0574-4

[B66] Shyamli P.S. Pradhan S. Panda M. Parida A. (2021). *De novo* whole-genome assembly of Moringa oleifera helps identify genes regulating drought stress tolerance. Front. Plant Sci. 12, 766999. doi: 10.3389/fpls.2021.766999 34970282 PMC8712769

[B67] Simão F. A. Waterhouse R. M. Ioannidis P. Kriventseva E. V. Zdobnov E. M. (2015). BUSCO: assessing genome assembly and annotation completeness with single-copy orthologs. Bioinformatics 31, 3210–3212. doi: 10.1093/bioinformatics/btv351 26059717

[B68] Stracke R. Werber M. Weisshaar B. (2001). The R2R3-MYB gene family in Arabidopsis thaliana. Curr. Opin. Plant Biol. 4, 447–456. doi: 10.1016/S1369-5266(00)00199-0 11597504

[B69] Suranjika S. Pradhan S. Nayak S. S. Parida A. (2022). *De novo* transcriptome assembly and analysis of gene expression in different tissues of moth bean (Vigna aconitifolia)(Jacq.) Marechal. BMC Plant Biol. 22, 198. doi: 10.1186/s12870-022-03583-z 35428206 PMC9013028

[B70] Wang G. Gao X. Wang X. Liu P. Guan S. L. Qi K.. (2022). Transcriptome analysis reveals gene associated with fruit size during fruit development in pear. Scientia Hortic. 305, 111367. doi: 10.1016/j.scienta.2022.111367

[B71] Wang Y. Liu H. Wang S. Li H. (2017). Genome-wide identification and expression analysis of the YUCCA gene family in soybean (Glycine max L.). Plant Growth Regul. 81, 265–275. doi: 10.1007/s10725-016-0203-x

[B72] Wang W. Wang Y. Chen T. Qin G. Tian S. (2023). Current insights into posttranscriptional regulation of fleshy fruit ripening. Plant Physiol. 192, 1785–1798. doi: 10.1093/plphys/kiac483 36250906 PMC10315313

[B73] Wu W. Cheng Z. Liu M. Yang X. Qiu D. (2014). C3HC4-type RING finger protein Nb ZFP1 is involved in growth and fruit development in Nicotiana benthamiana. PloS One 9, e99352. doi: 10.1371/journal.pone.0099352 24901716 PMC4047095

[B74] Xanthopoulou A. Ganopoulos I. Psomopoulos F. Manioudaki M. Moysiadis T. Kapazoglou A.. (2017). *De novo* comparative transcriptome analysis of genes involved in fruit morphology of pumpkin cultivars with extreme size difference and development of EST-SSR markers. Gene 622, 50–665. doi: 10.1016/j.gene.2017.04.035 28435133

[B75] Xanthopoulou A. Montero-Pau J. Picó B. Boumpas P. Tsaliki E. Paris H. S.. (2021). A comprehensive RNA-Seq-based gene expression atlas of the summer squash (Cucurbita pepo) provides insights into fruit morphology and ripening mechanisms. BMC Genomics 22, 341. doi: 10.1186/s12864-021-07683-2 33980145 PMC8114506

[B76] Xu J. Xiong W. Cao B. Yan T. Luo T. Fan T.. (2013). Molecular characterization and functional analysis of “fruit-weight2. 2-like” gene family in rice. Planta 238, 643–655. doi: 10.1007/s00425-013-1916-y 23793979

[B77] Yang L. Liu H. Zhao J. Pan Y. Cheng S. Lietzow C. D.. (2018). LITTLELEAF (LL) encodes a WD40 repeat domain-containing protein associated with organ size variation in cucumber. Plant J. 95, 834–847. doi: 10.1111/tpj.13991 29901823

[B78] Yang X. Y. Wang Y. Jiang W. J. Liu X. L. Zhang X. M. Yu H. J.. (2013). Characterization and expression profiling of cucumber kinesin genes during early fruit development: revealing the roles of kinesins in exponential cell production and enlargement in cucumber fruit. J. Exp. Bot. 64, 4541–4557. doi: 10.1093/jxb/ert269 24023249 PMC3808332

[B79] Yuste-Lisbona F. J. Fernández-Lozano A. Pineda B. Bretones S. Ortíz-Atienza A. García-Sogo B.. (2020). ENO regulates tomato fruit size through the floral meristem development network. Proc. Natl. Acad. Sci. 117 (14), 8187–8195. doi: 10.1073/pnas.1913688117 32179669 PMC7148573

[B80] Zhang H. Tan J. Zhang M. Huang S. Chen X. (2020). Comparative transcriptomic analysis of two bottle gourd accessions differing in fruit size. Genes 11, 359. doi: 10.3390/genes11040359 32230807 PMC7230174

[B81] Zhang C. Tanabe K. Tani H. Nakajima H. Mori M. Sakuno E. (2007). Biologically active gibberellins and abscisic acid in fruit of two late-maturing Japanese pear cultivars with contrasting fruit size. J. Am. Soc. Hortic. Sci. 132, 452–458. doi: 10.21273/JASHS.132.4.452

[B82] Zhang H. Wang H. Yi H. Zhai W. Wang G. Fu Q. (2016). Transcriptome profiling of Cucumis melo fruit development and ripening. Horticulture Res. 3. doi: 10.1038/hortres.2016.14 PMC4847005 27162641

[B83] Zhang L. Xu Y. Li Y. Zheng S. Zhao Z. Chen M.. (2024). Transcription factor CsMYB77 negatively regulates fruit ripening and fruit size in citrus. Plant Physiol. 194 (2), 867–883. 37935634 10.1093/plphys/kiad592

[B84] Zhang Y. S. Xu Y. Xing W. T. Wu B. Huang D. M. Ma F. N.. (2023). Identification of the passion fruit (Passiflora edulis sims) MYB family in fruit development and abiotic stress, and functional analysis of PeMYB87 in abiotic stresses. Front. Plant Sci. 14, 1124351. 37215287 10.3389/fpls.2023.1124351 PMC10196401

[B85] Zhang H. Wei L. Miao H. Zhang T. Wang C. (2012). Development and validation of genic-SSR markers in sesame by RNA-seq. BMC Genomics 13, 1–11. doi: 10.1186/1471-2164-13-316 22800194 PMC3428654

[B86] Zhao X. Muhammad N. Zhao Z. Yin K. Liu Z. Wang L.. (2021). Molecular regulation of fruit size in horticultural plants: A review. Scientia Hortic. 288, 1103535. doi: 10.1016/j.scienta.2021.110353

