# Supplementary figures and images for: Transcriptomics reveal useful resources for examining fruit development and variation in fruit size in Coccinia grandis
Source: Front Plant Sci. 2024 May 28;15:1386041. doi: 10.3389/fpls.2024.1386041 (PMC11165041; doi:10.3389/fpls.2024.1386041)

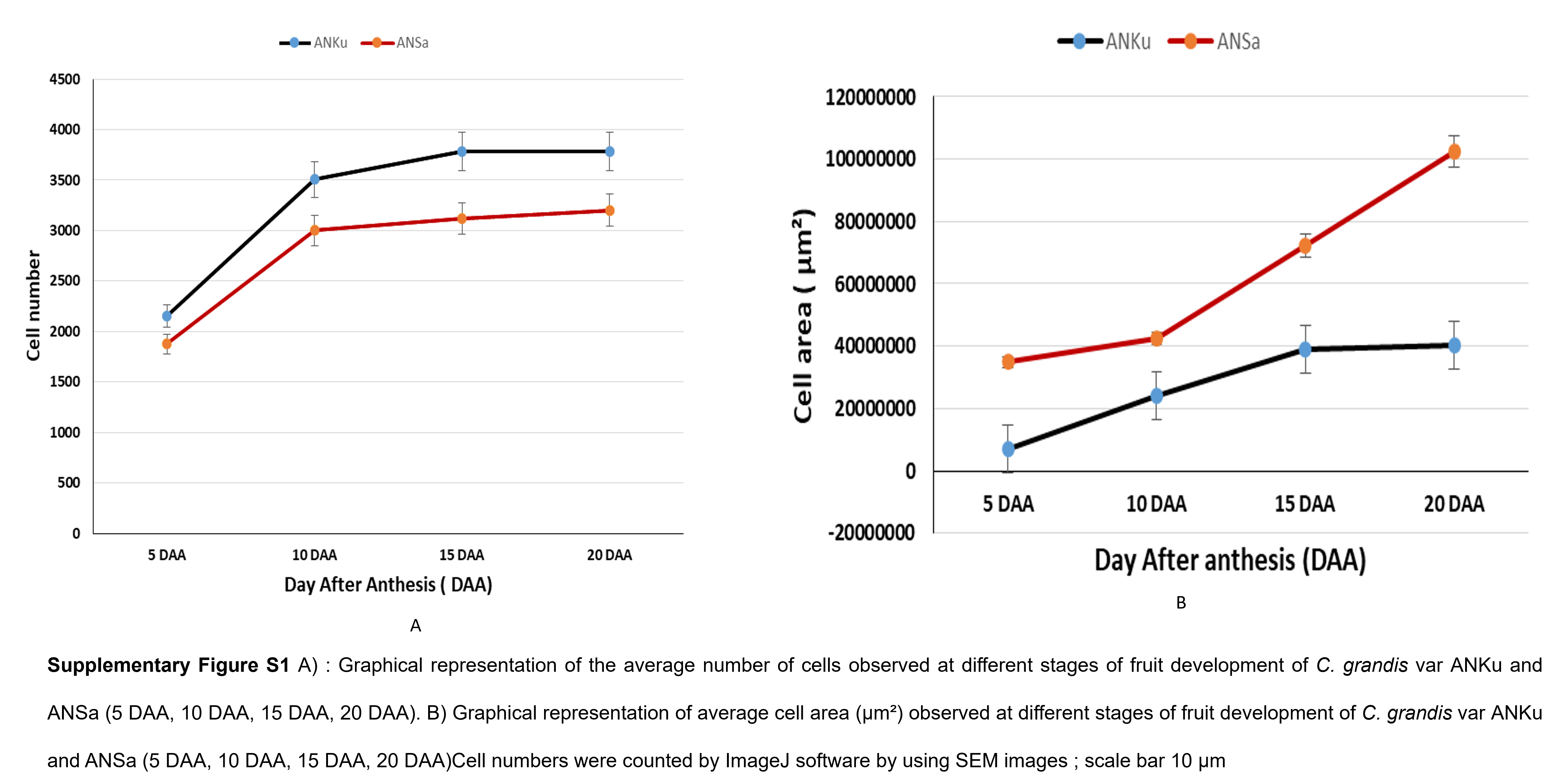

Supplement: Supplementary file 9 [file Image_1.tif]

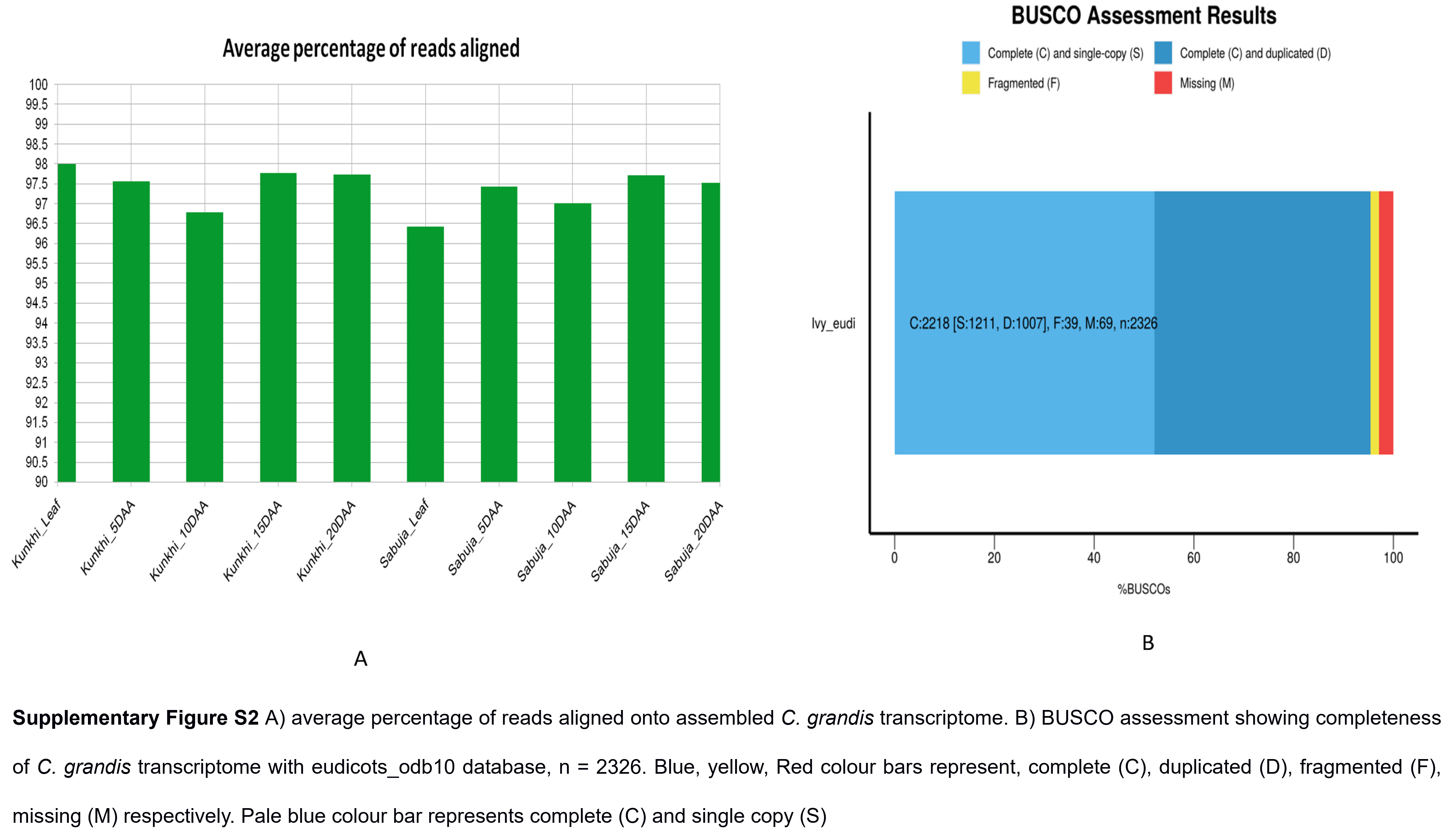

Supplement: Supplementary file 10 [file Image_2.tif]

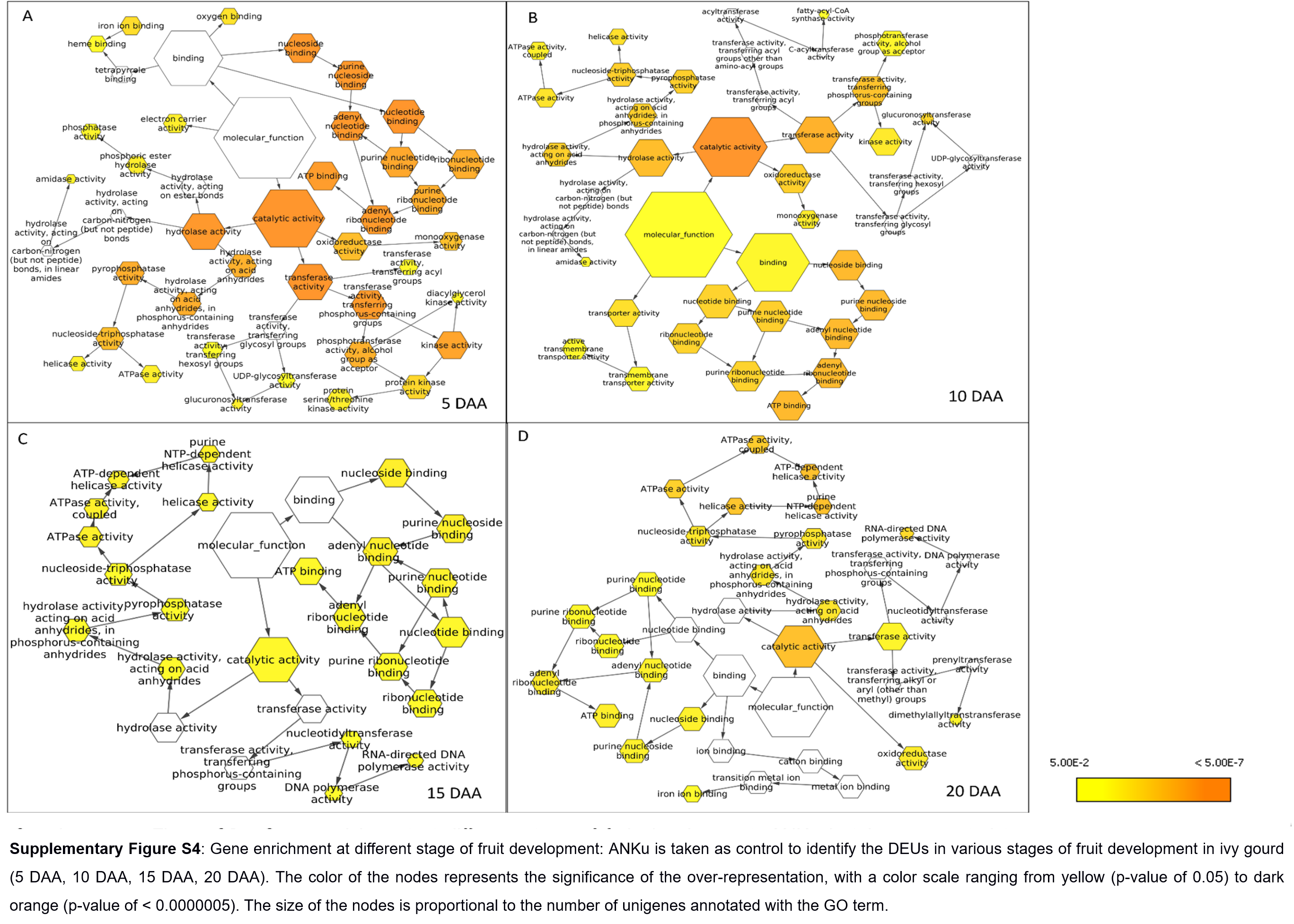

Supplement: Supplementary file 12 [file Image_4.tif]

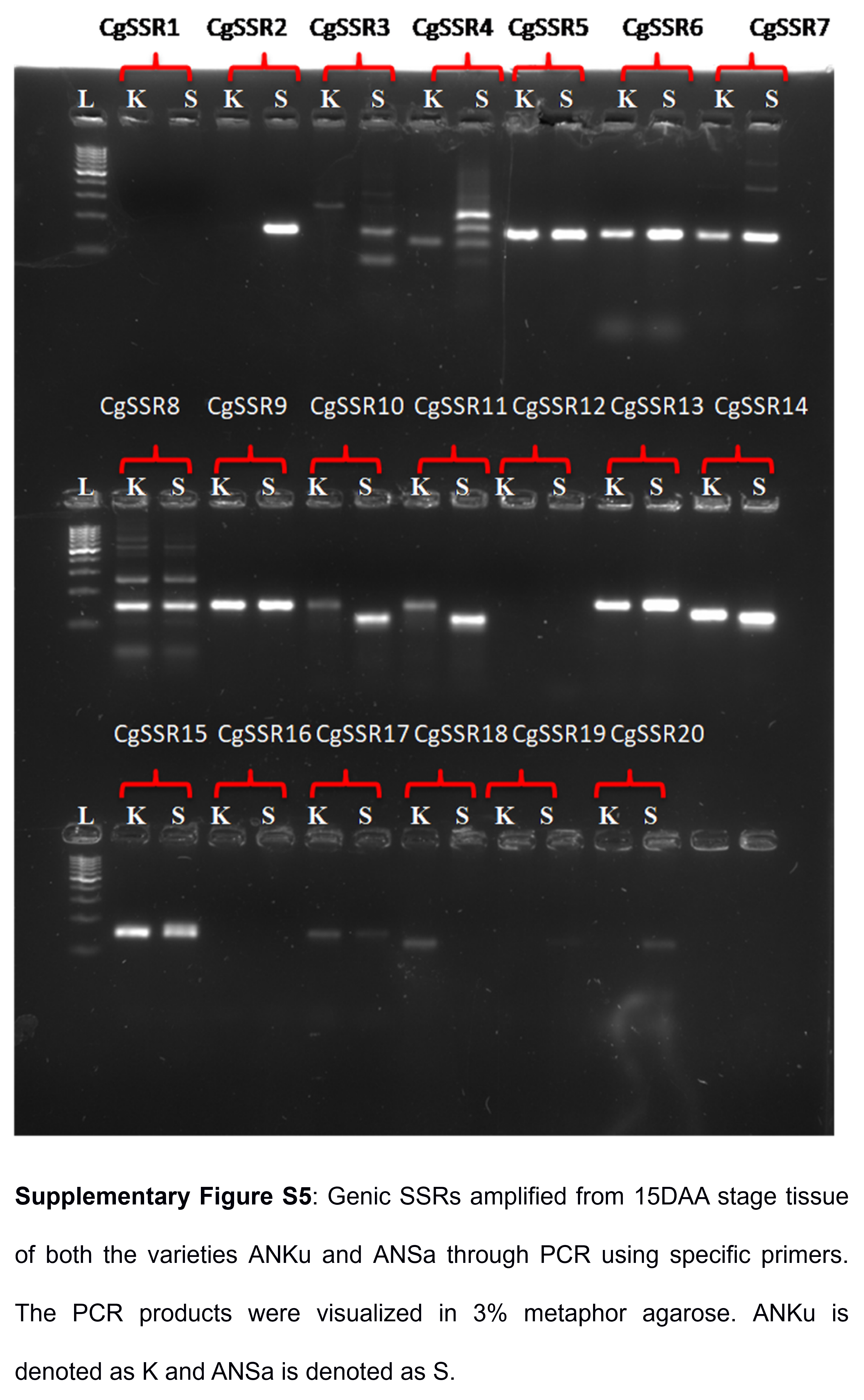

Supplement: Supplementary file 13 [file Image_5.tif]
